# Characterization of tomato protein kinases embedding guanylate cyclase catalytic center motif

**DOI:** 10.1038/s41598-020-61000-7

**Published:** 2020-03-05

**Authors:** Hafizur Rahman, Xin-Yao Wang, You-Ping Xu, Yu-Han He, Xin-Zhong Cai

**Affiliations:** 1 Institute of Biotechnology, College of Agriculture and Biotechnology, Zhejiang University, Hangzhou, 310058 China; 2 Center of Analysis and Measurement, Zhejiang University, Hangzhou, 310058 China

**Keywords:** Plant immunity, Plant stress responses

## Abstract

Guanylate cyclases (GCs) are enzymes that catalyze the reaction to produce cyclic GMP (cGMP), a key signaling molecule in eukaryotes. Nevertheless, systemic identification and functional analysis of GCs in crop plant species have not yet been conducted. In this study, we systematically identified GC genes in the economically important crop tomato (*Solanum lycopersicum* L.) and analyzed function of two putative tomato GC genes in disease resistance. Ninety-nine candidate GCs containing GC catalytic center (GC-CC) motif were identified in tomato genome. Intriguingly, all of them were putative protein kinases embedding a GC-CC motif within the protein kinase domain, which was thus tentatively named as GC-kinases here. Two homologs of Arabidopsis PEPRs, SlGC17 and SlGC18 exhibited *in vitro* GC activity. Co-silencing of *SlGC17* and *SlGC18* genes significantly reduced resistance to tobacco rattle virus, fungus *Sclerotinia sclerotiorum*, and bacterium *Pseudomonas syringae* pv. *tomato* (*Pst*) DC3000. Moreover, co-silencing of these two genes attenuated PAMP and DAMP-triggered immunity as shown by obvious decrease of flg22, chitin and AtPep1-elicited Ca^2+^ and H_2_O_2_ burst in *SlGC*-silenced plants. Additionally, silencing of these genes altered the expression of a set of Ca^2+^ signaling genes. Furthermore, co-silencing of these GC-kinase genes exhibited stronger effects on all above regulations in comparison with individual silencing. Collectively, our results suggest that GC-kinases might widely exist in tomato and the two *SlPEPR-GC* genes redundantly play a positive role in resistance to diverse pathogens and PAMP/DAMP-triggered immunity in tomato. Our results provide insights into composition and functions of GC-kinases in tomato.

## Introduction

Guanosine 3′,5′-cyclic monophosphate (cGMP) is a well known intracellular second messenger that regulates a broad range of physiological responses in animals, fungi and prokaryotes. However, the existence and function of cGMP in higher plants has been controversial for many years^1^. The level of cGMP in higher plants is much lower than that in animals^2^. Nevertheless, with the improvement of cGMP determination methods, it has been generally recognized that cGMP is present in plants^3–5^. Furthermore, cGMP has been found to be involved in many molecular and cellular events such as protein phosphorylation^6,7^, transcription regulation^8,9^, hormone functioning^6,10,11^ and cation flux regulation^8,12,13^, as well as a variety of developmental processes such as development of adventitious roots^14^, chloroplast development^15^, seed germination^16^, stomatal movement^17^, reorientation of pollen tube^18^, anthocyanin and flavonoid biosynthesis^9^, and photoperiodic flower induction^19^. Additionally, cGMP is important in response to biotic stresses^20–24^ and abiotic stresses^25,26^. Moreover, cGMP appears to act on components such as cyclic nucleotide-gated ion channels (CNGCs), cGMP-dependent kinase G (PKG) and cyclic nucleotide phosphodiesterases (PDEs) to modulate plant responses^22,23,27–31^.

While cGMP is increasingly accepted as an important signaling molecule in plant, the mechanism underlying cGMP metabolism in plant remains poorly understood^1^. In animal, guanylate cyclase (GC) catalyzes the synthesis of cGMP from guanosine 5′-triphosphate (GTP)^32^. However, protein sequences with high similarity to known GCs have not yet been identified in higher plant; although GCs have been reported in lower plants^1,33,34^. Nonetheless, searching analyses with a motif deduced from representatives of annotated catalytic domains of GCs from prokaryotes and eukaryotes ([RKS] [YFW] [CTGH] [VIL] [FV] G [DNA] x [VIL] x{4} [KR]) demonstrated that sequences carry the motif of functional amino acid (AA) residues of the GC catalytic center (GC-CC) do exist in plants^35–38^. In this motif, the AA at position 1 forms the hydrogen bond with the guanine, the residue at position 3 confers substrate specificity for GTP, the AA at position 14 stabilizes the transition state from GTP to cGMP, while two or three AAs away from the C-terminal end of the motif is the residue that interacts with the Mg^2+^/Mn^2+^ ions^35,36^. Searches using this motif led to identification of several *Arabidopsis thaliana* proteins that exhibit GC activity *in vitro*^35,37^. These include; *A. thaliana* guanylate cyclase 1 (AtGC1), brassinosteroid receptor (AtBRI1), wall associated kinase-like 10 (AtWAKL10), Pep receptor 1 (AtPEPR1), phytosulfokine (PSK) receptor (AtPSKR1), nitric oxide (NO) dependent guanylate cyclase 1 (NOGC1), and plant natriuretic peptide receptor (AtPNP-R1)^22,38–43^. The conserved 14-AA GC-CC motif of these functionally confirmed AtGCs is [KS] [YF] [GCS] [VIL] [VILFG] [DVIL] [VILADG] [EPVIL] [DVIL] [TVIL] [WST] [PDRG] [KEG] [KR]^35^. The GC activity of AtBRI1 was not confirmed in studies examining its crystal structure but was confirmed recently by detecting the activity in the whole cytoplasmic domain of AtBRI1^44,45^. Additionally, two GCs, HpGC1 and HpPEPR1 were identified from bulbous plant *Hippeastrum hybridum*^46,47^.

The potential GC proteins identified to date are invovled in diverse biological processes including plant growth and defense against pathogens. For instance, AtBRI1, AtPSKR1 and AtPNP-R1 are the receptor of the peptide hormone BR, PSK and AtPNP-A, respectively^43,48,49^, while AtPEPR1 is the receptor of Peps which elicit DAMP-triggered immunity^22,23^. Notably, GC-CC motif exists within the kinase domain of several leucine rich repeat (LRR)-receptior like kinases (LRR-RLKs) including AtBRI1, AtWAKL10, AtPSKR1 and AtPEPR1. Interestingly, the kinase activity of AtBRI1 is required to activate GC to produce cGMP, which stimulates downstream phosphorylation signals^45^. This finding highlights the important role of GC embedded in the knase domain of LRR-RLKs in their signaling function.

To date genome-wide identification of plant GC has only been conducted in Arabidopsis^35^. In this study, we systematically identified candidate GCs in the genome of the economically important crop tomato (*Solanum lycopersicum* L.) and analyzed the function of two of them in disease resistance. We demonstrated that tomato genome harbors 99 putative GC-kinases and two tomato homologs of AtPEPR1 redundantly and positively regulate various types of defense and resistance. Our results provide first insights into the geonome-wide composition and function of GCs in crop species.

## Methods

### Identification of GC proteins in tomato genome

BLASTp search was performed against tomato (*Solanum lycopersicum* L.) genome in Phytozome (http://www.Phytozome.net) and NCBI (http://www.ncbi.nlm.nih.gov/) using known Arabidopsis GC proteins as queries. Meanwhile, pattern-hit initiated BLAST (phi-BLAST) search was conducted in NCBI database using the plant GC-specific GC-CC motif [KS] [YF] [GCS] [VIL] [VILFG] [DVIL] [VILADG] [EPVIL] [DVIL] [TVIL] [WST] [PDRG] [KEG] [KR] x{2,3} [DHSE]^35^ as query. All retrieved non-redundant sequences were collected and subjected to GC-CC motif prediction analysis using GCPred tool^50^ and domain analysis using the Pfam (http://pfam.sanger.ac.uk/) and Conserved Domain Database (CDD) (http://www.ncbi.nlm.nih.gov/Structure/cdd/wrpsb.cgi/) programs. The predicted GC-CC motif or full length amino acid sequences were aligned using ClustalW2 program (http://www.ebi.ac.uk/Tools/msa/clustalw2/) with default settings and were viewed by GeneDoc program.

### Phylogenetic analysis of tomato and Arabidopsis candidate GCs

Multiple sequence alignments of the full-length candidate GC protein sequences from tomato and Arabidopsis were conducted using clustalW2 program^51^. The phylogenetic tree was constructed using MEGA 5.0^52^ with maximum likelihood (ML) method and a bootstrap test was performed with 1,000 replicates.

### Virus-induced gene silencing (VIGS) analyses

To ensure the specificity to target an individual gene member, gene primers for the VIGS target fragments of *SlGC17* and *SlGC18* were designed from the gene specific regions. The target fragments of *SlGC* genes were amplified by PCR using gene specific primers (Supplementary Table S1) with *Bam*HI (ggatcc) and *Eco*RI (gaattc) restriction sites for forward and reverse primers, respectively. These fragments were cloned and ligated into the TRV-based VIGS vector pYL156, and were subsequently electroporated into *Agrobacterium tumefaciens* strain GV3101 for VIGS analyses. Agro-inoculation were conducted with vacuum-infiltration delivery approach as previously described^53,54^ except that recombinant pYL156 with insertion of an eGFP fragment instead of an empty pYL156 was used as control to alleviate viral symptom^55^. For co-silencing, suspensions of Agrobacterium carrying pYL156::*SlGC17* and pYL156::*SlGC18*, respectively, were equally mixed before infiltration into tomato leaves. VIGS was performed in normal and aequorin gene transgenic tomato seedlings.

### Plant inoculation and disease resistance analysis

At three weeks post agro-infiltration, the tomato plants were inoculated with a variety of pathogens including *Sclerotinia sclerotiorum*, *Pseudomonas syringae* pv*. tomato* (*Pst*) DC3000 and *Xanthomonas oryzae* pv*. oryzae* (*Xoo*). The inoculation and disease resistance evaluation including *Sclerotinia sclerotiorum*-caused lesion size measurement and bacterial number counting in *Pst* DC3000- and *Xoo*-inoculated leaf areas were conducted as described^24,56^.

### ROS detection

For *in situ* H_2_O_2_ detection, *Pst* DC3000 and *Xoo* inoculated leaves of tomato plants were detached and stained with 3,3-diamino benzidine hydrochloride (DAB) (1 mg/mL) as previously described^57^. For quantitative analyses, tomato leaf disks of 3 mm at diameter were dipped in 200 μl of distilled water in a 96-well plate in dark over night. Water was replaced with 200 μl solution containing 100 μM luminol (Sigma-Aldrich) and 1 μg of horseradish peroxidase. The H_2_O_2_ elicitors; bacterial PAMP flg22 (100 nM), fungal PAMP chitin (100 μg mL^−1^), DAMP AtPep1 (10 nM) and *Pst* DC3000 bacterial cells (OD_600_ = 0.1) were added and immediately H_2_O_2_ were measured for 35 min as luminescence using a Microplate Luminometer (TITERTEK BERTHOLD, Germany).

### Calcium detection

Transient increase of cytosolic Ca^2+^ concentration was monitored in the Aequorin gene transgenic tomato line^58^. Leaf discs dispatched on a 96-well plate were incubated overnight in 12.5 mM coelenterazin (LUX Innovate). Before measurement, the solution was removed and 100 mL of assay solution containing a PAMP or DAMP or *Pst* DC3000 bacterial cells as described above was added to the wells. Luminescence was measured using a Microplate Luminometer (TITERTEK BERTHOLD, Germany).

### Gene expression analyses by real time PCR

Quantitative real time PCR (qRT-PCR) analyses and consequent statistical data analyses were conducted as described^59^. A tomato rRNA gene was used as internal control^56^. The gene-specific primers used in qRT-PCR analyses were listed in Supplementary Table S1.

### Detection of *in vitro* catalytic activity of SlGC17_724-1105_ and SlGC18_788-1104_ to generate cGMP

The cDNA sequences corresponding to the intracellular domain of SlGC17 and SlGC18 proteins (SlGC17_724–1105_ and SlGC18_788–1104_) were amplified from tomato cv. Moneymaker through RT-PCR using gene specific primers (SlGC17_724–1105_-F: 5′-GGCAAAATACGGAACCGCA-3′; SlGC17_724–1105_-R: 5′-CTGGTTGCTGGTGCTATAGCTAAC-3′; SlGC18_788–1104_-F: 5′-CGCAAAAGTTCTGGGAAAGG-3′; SlGC18_788–1104_-R: 5′-GTACTTGCTTCGTATACTCGAACTTGA-3′), which were then cloned into prokaryotic expression vector pET32a and were expressed in *Escherichia coli* BL21 (DE3) pLysS (Trans-Gen Biotech, Beijing, China). His-tagged SlGC17_724–1105_ and SlGC18_788–1104_ recombinant proteins were affinity purified using His antibody following manufacturer’s instructions (TaKaRa, Japan).

The *in vitro* GC activity of purified SlGC17_724–1105_ and SlGC18_788–1104_ was manifested as the produced cGMP content measured using the Amersham Biosciences cGMP enzyme immunoassay Biotrak system (GE Healthcare, USA, code RPN226). Ten μg of each protein was incubated in 100 μl of 50 mm Tris-HCl (pH 8.0) containing 2 mm isobutylmethylxanthine (IBMX), 5 mm MgCl_2_ and 1 mm GTP. Background cGMP levels in the reaction mixture were measured without addition of protein. After incubation for 5, 10 and 15 min at 24 °C, respectively, the reaction was terminated by addition of 10 mM EDTA. Tubes were then boiled for 3 min, cooled on ice for 2 min and centrifuged at 2300 × g for 3 min. The supernatant was collected for cGMP content measurement based on highly specific anti-cGMP antibody using the system mentioned above following the Protocol 2 as described in the supplier’s manual.

In order to verify the enzyme immunoassay result, the produced cGMP in the reactions was further identified by mass spectrometry (MS) assay. MALDI-TOF-MS analyses were performed using a Bruker UltrafleXtreme mass spectrometer equipped with a laser (355 nm, 2000 Hz) (Bruker Daltonics, Bremen, Germany). 3-Hydroxy picolinic acid (3-HPA) was used as matrix. One μl matrix was spotted onto the Ground steel and dried in air. One μl reaction mixture or cGMP standard (Sigma-Aldrich, USA, G7504) was then spotted onto the dried matrix and dried in air, which was subjected to MS analyses. Reflector positive mode was used for detection. Spectra data were collected by FlexControl software and processed using the FlexAnalysis software.

### Statistical analysis

For VIGS and resistance evaluation analyses, at least 10 plants per pathogen for each gene were used in each experiment. For H_2_O_2_ and Ca^2+^ detection as well as gene expression analyses, 10 leaves from 10 plants for each treatment were collected in each experiment. All experiments were conducted three times independently. The data from quantitative analyses were statistically analyzed using SPSS software (Version 19.0, IBM, USA) and represent means ± standard error (SE) of three independent experiments. Significant difference is analyzed by Duncan’s multiple range test (DMRT, *p* < 0.05).

## Results

### Identification of candidate GCs in tomato genome

**Figure 1:**
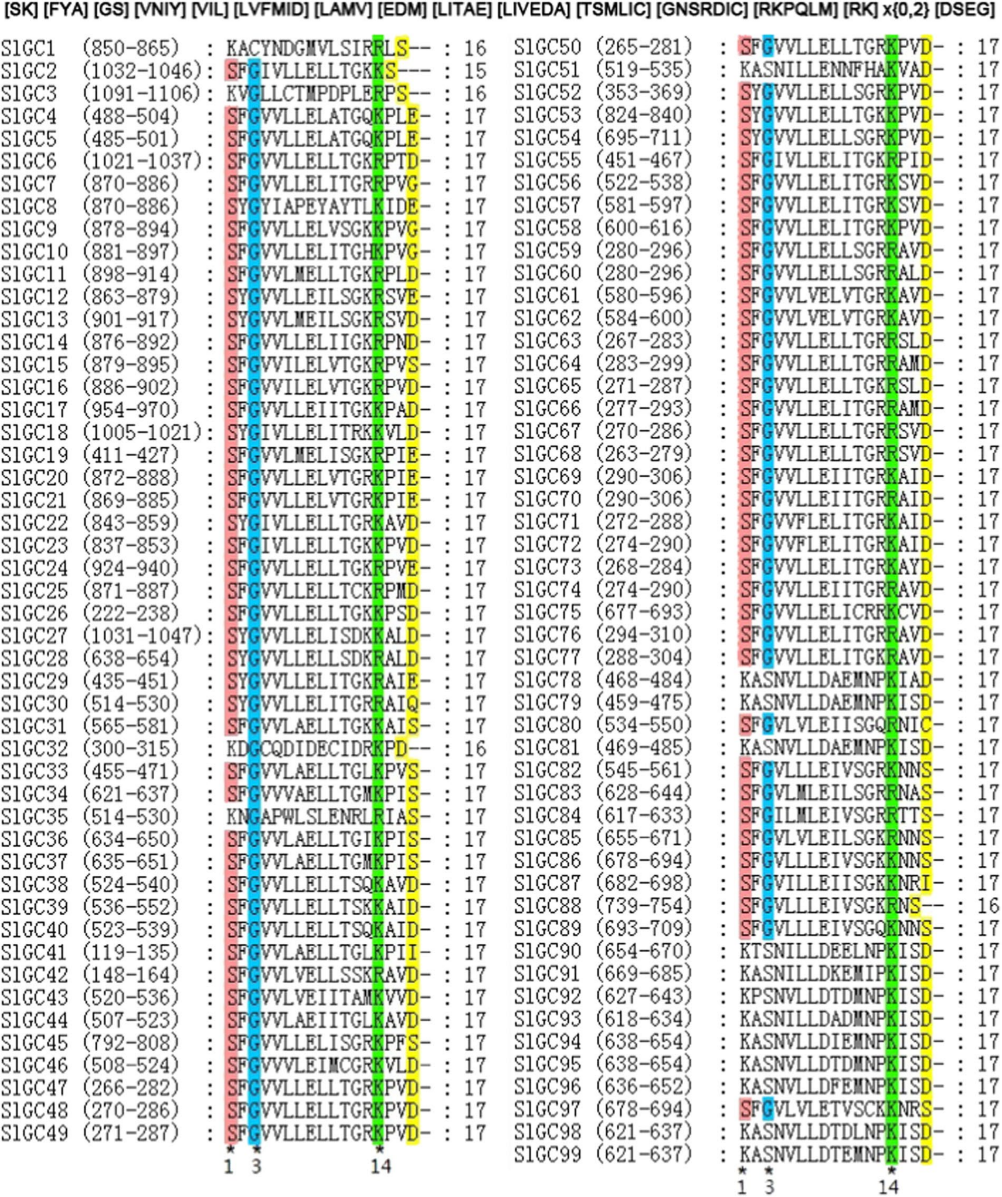
Alignment of the GC catalytic center (GC-CC) regions of the 99 candidate GCs identified in tomato. The GC-CC motif conserved in tomato GCs are deduced from the alignment and shown at top. The most conserved amino acid residues required for GC catalysis function are highlighted in different colors and their positions are indicated at bottom of the alignment.

To identify GC candidates in tomato, two ways of BLAST searches were performed. First, BLASTp search was conducted against tomato genome in Phytozome and NCBI using known Arabidopsis GC proteins as queries. Meanwhile, pattern-hit initiated BLAST (phi-BLAST) was carried out against tomato genome in NCBI using the plant GC-specific GC catalytic center (GC-CC) motif ([KS] [YF] [GCS] [VIL] [VILFG] [DVIL] [VILADG] [EPVIL] [DVIL] [TVIL] [WST] [PDRG] [KEG] [KR] x{2,3} [DHSE])^35^ as query. Resultantly, 99 non-redundant sequences were retrieved. These sequences were then further subjected to prediction for GC-CC motif including the amino acid implicated in co-factor (Mg^2+^/Mn^2+^) binding using GCPred tool^50^. All sequences except sequence #41 were predicted to contain one to several potential GC-CC motifs (Supplementary Table S2). When the sequence #41 was aligned with AtGCs, a GC-CC motif was also predicted (Supplementary Table S3). Alighment of the 98 GC-CC motifs predicted using GCPred with the highest confidence together with the one for sequence #41 predicted from alignment analysis revealed that all these motifs contained [SK]1, [GSC]3 and [KR]14, the three AAs associated with catalysis function in the plant GC-specific GC-CC motif, and thus the 99 sequences were recognized as tomato GC candidates (Fig. 1). The 99 tomato GC candidates were listed in Supplementary Table S3.

### The GC-CC motif was embedded within the kinase domain of the identified tomato GC candidates

**Figure 2:**
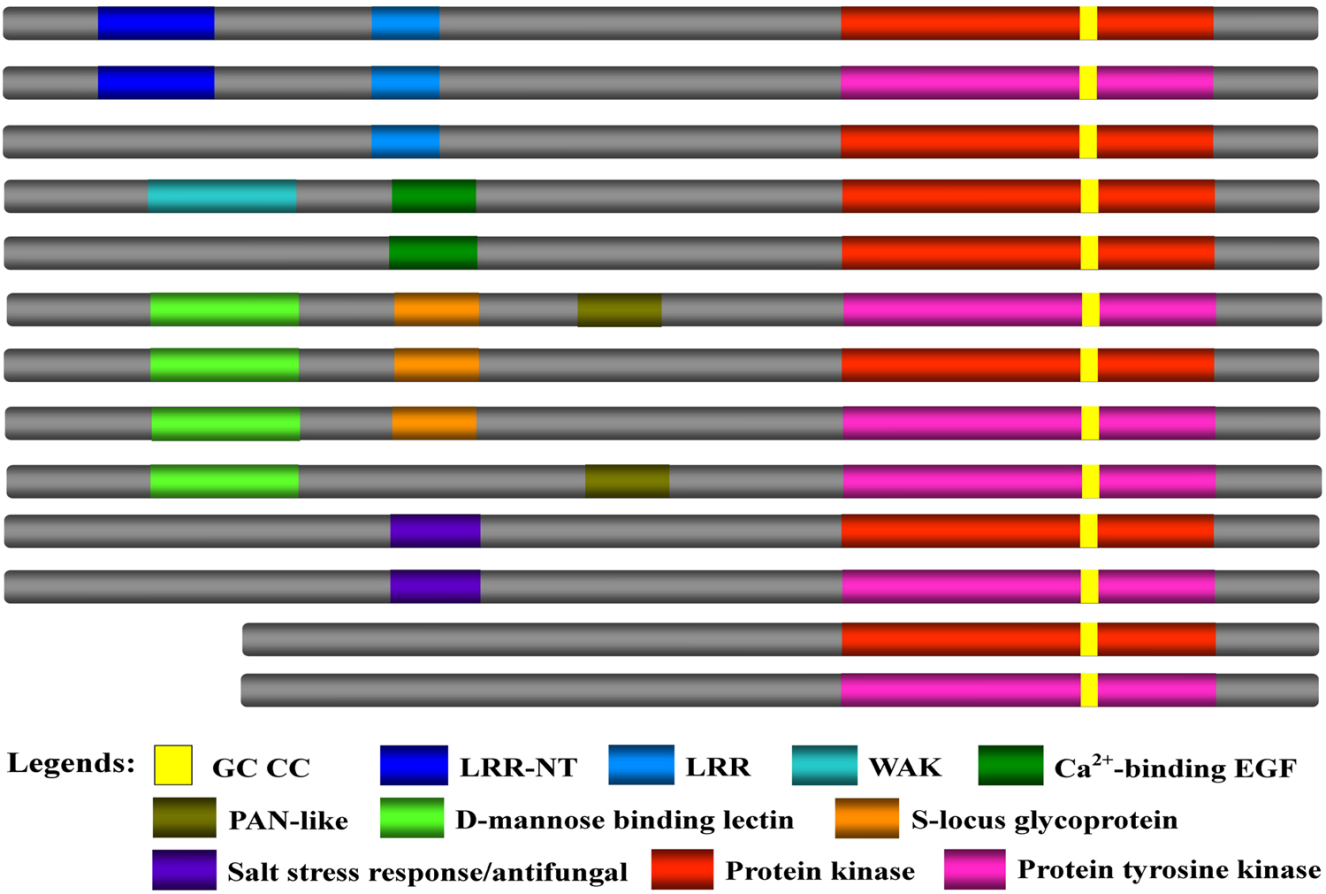
Schematic representation of domain organization of tomato candidate GCs. Domains are indicated in different colors. The GC-CC motif was encapsulated within the kinase domain in all 99 putative tomato GCs.

Domain composition analyses showed that all the 99 tomato GC candidates contained a protein kinase domain, either Ser/Thr-type or Tyr-type (Fig. 2), demonstrating that they were likely protein kinases. The putative GC protein sequences represented 13 types of domain combinations of 10 different domains. The domains included LRR-NT, LRR, WAK, Ca^2+^-binding EGF (CB_EGF), PAN-like (PANL), D-mannose binding lectin (MB_Lectin), S-locus glycoprotein (SGP), salt stress response/antifungal (SSR/AF), Pkinase and Pkinase-Tyrosine (PKinase_Tyr) (Fig. 2). Out of the 99 putative tomato GCs, 27 were LRR-Pkinase/Pkinase_Tyr proteins, 6 were CB_EGF-Pkinase proteins with one carrying an extra WAK domain in the N-terminus, 18 were MB_Lectin-Pkinase_Tyr/Pkinase proteins with additionally one of or both SGP and PANL domains, 5 were SSR/AF-Pkinase/Pkinase_Tyr proteins, while 26 and 17 solely possessed a Pkinase or Pkinase_Tyr domain, respectively (Supplementary Table S3). These results indicate that GC-CC tends to combine with other domains especially protein kinase domains to form multifunctional proteins.

Intriguingly, the GC-CC motif was embedded within the kinase domain for all 99 sequences (Fig. 2), indicating that kinase domain is the preferable sequence to hide the GC-CC motif and that the GC and kinase dual functional proteins are widely present in the tomato genome. These GC-CC-motif embedded kinases were therefore abbreviated as GC-kinases hereafter in this paper.

The GC-CC motif region of the 99 putative tomato GC-kinases was aligned. This alignment revealed a tomato GC-CC motif as ([SK] [FYA] [GS] [VNIY] [VIL] [LVFMID] [LAMV] [EDM] [LITAE] [LIVEDA] [TSMLIC] [GNSRDIC] [RKPQLM] [RK] x{0,2} [DSEG]), where the amino acids in a certain position were listed in the order of frequency from high to low and existed in at least two SlGCs) (Fig. 1). Comparison of this motif with the one for plant as described above demonstrated that the tomato GC-CC motif was as conserved as the plant GC-CC motif for the functionally associated AAs at positions 1, 3 and 14, while was more relaxed at some other positions. It was notable that A2, N4, D8, T9, [ED]10, M11, [GN]12, [RPQL]13 appearred frequently in predicted tomato GCs but not included in plant GC-CC motif. This result suggests that the plant GC-CC motif needs to be relaxed for these positions when used for plant GC prediction through phi-BLAST search.

### Phylogenetic relationship between tomato and Arabidopsis GCs

**Figure 3:**
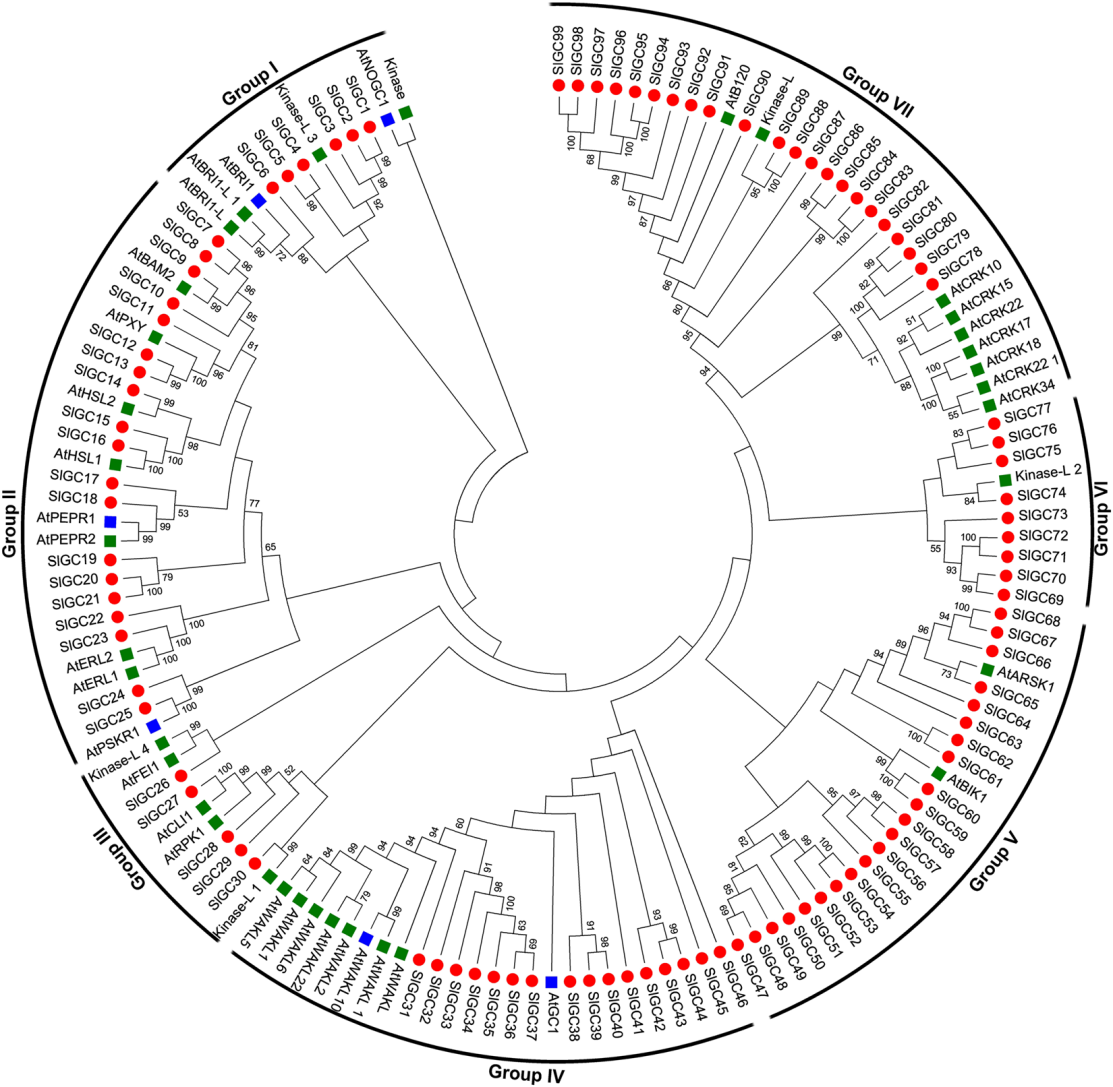
Phylogenetic tree of tomato and Arabidopsis candidate GCs. The phylogenetic tree was constructed using MEGA 5.0 with maximum likelihood (ML) method and a bootstrap test was performed with 1,000 replicates. Tomato candidate GCs are marked with a solid red circle while Arabidopsis candidate GCs and functionally confirmed GCs are indicated with a green and a blue square box, respectively.

To examine the phylogenetic relationships and functional associations of candidate tomato GCs, a total of 140 plant GC protein sequences including 99 tomato and 41 Arabidopsis GC candidates were used to construct a phylogenetic tree based on maximum likelihood (ML) method (Fig. 3). The phylogenetic analysis indicated that 99 tomato GCs clustered into seven groups (group I-VII). Six tomato GCs (SlGC1-SlGC6) and six Arabidopsis GCs gathered in group I, which included experimentally confirmed GCs AtNOGC1 and AtBRI1^39,42^. Nineteen tomato GCs (SlGC7-SlGC25) and 9 Arabidopsis GCs formed group II, which included AtPEPR1 and AtPSKR1, two well studied GCs^22,41^. Five tomato GCs (SlGC26-SlGC30) and 5 Arabidopsis GCs clustered into group III. Sixteen tomato GCs (SlGC31-SlGC46) and 9 Arabidopsis GCs comprised group IV, which included functional GCs AtWAKL10 and AtGC1^38,39^ and seven other AtWAKL kinases. In addition, group V was comprised of 22 tomato GCs (SlGC47-SlGC68) and 2 Arabidopsis GCs (AtBIK1 and AtARSK1), group VI constituted of 9 tomato GCs (SlGC69-SlGC77) and one Arabidopsis GC (kinase-L2) while group VII consisted of 22 tomato GCs (SlGC78-SlGC99) and 9 Arabidopsis GCs among which were 7 AtCRK proteins (Fig. 3).

Interestingly, SlGC6 and AtBRI1, SlGC17/SlGC18 and AtPEPRs, as well as SlGC24/SlGC25 and AtPSKR1 clustered in the same clade of group I, II and III, respectively, in the phylogenetic tree (Fig. 3). Full-length amino acid sequence alignment further showed that they shared with high sequence homology (Supplementary Fig. S1). These results indicate that these putative tomato GCs may be orthologs of the corresponding Arabidopsis GCs.

### Silencing of putative tomato PEPR-GC genes *SlGC17* and *SlGC18* altered resistance to diverse pathogens

The phylogenetic and sequence alignment analyses indicated that SlGC6 and SlGC17/SlGC18 are the homologs of the functionally confirmed GCs AtBRI1 and AtPEPR1, respectively (Fig. 3, Supplementary Fig. S1). Moreover, AtPEPR1 plays an important role in plant disease resistance as a receptor of the Pep elicitors^22,23^. These findings prompted us to investigate whether these putative *SlPEPR-GCs* function in calcium regulated plant disease resistance through virus-induced gene silencing (VIGS) analyses.

**Figure 4:**
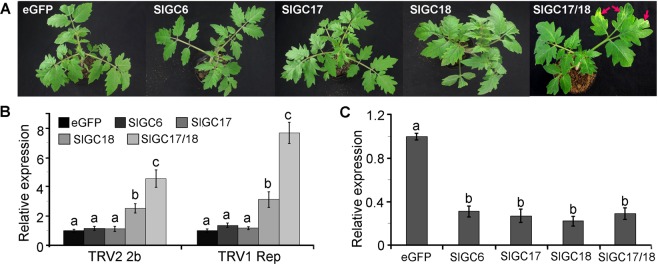
Silencing of the putative tomato PEPR-GC genes *SlGC17* and *SlGC18* reduced resistance to TRV. Individual VIGS for three SlGC genes encoding a homolog of AtBRI1 (SlGC6) and two homologs of AtPEPR (SlGC17 and SlGC18) and co-silencing of SlGC17 and SlGC18 (SlGC17/18) were conducted. (**A**) Phenotypes of the SlGC-silenced plants. Red arrows indicate severe TRV viral mosaic symptoms in *SlGC17* and *SlGC18* co-silenced plants. Photograph were taken at three weeks post agro-inoculation. (**B**) Detection of transcripts of the TRV1 replicase and TRV2 2b genes in SlGC-silenced plants by qRT-PCR. (**C**) Evaluation of gene silencing efficiency. Expression levels of the SlGC genes in SlGC-silenced tomato plants were examined by qRT-PCR. Data represent mean ± SE of three independent experiments. The small letters indicate the signicant difference between expression levels in silenced plants and those in the eGFP control plants (*p* < 0.05, DMRT).

Gene specific target sequences of *SlGC6*, *SlGC17* and *SlGC18* were amplified and inserted into the tobacco rattle virus (TRV)-based VIGS vector pYL156 for silencing analyses, while non-silenced eGFP fragment-inserted recombinant pYL156 vector was used as a negative control^55^. To examine the functional redundancy of *SlGC17* and *SlGC18*, co-silencing for both genes was analyzed in addition to individual gene silencing. At three weeks after agro-infiltration, the tomato plants for co-silencing of *SlGC17* and *SlGC18* exhibited clear mosaic symptoms in leaves; those for *SlGC18* silencing displayed mild mosaic symptoms, while those for *SlGC6* and *SlGC17* individual silencing grew normally, as observed for control eGFP plants (Fig. 4A). Gene expression analysis revealed that transcripts of TRV_1_ replicase gene accumulated over 3 and 7 folds higher, while those of TRV_2_ 2b gene were near 3 and 5 folds higher in *SlGC18*-silenced and *SlGC17/SlGC18*-co-silenced plants, respectively, in comparison to eGFP control plants (Fig. 4B).

To clarify whether the *SlGC* genes had been efficiently silenced in the agro-infiltrated tomato plants, transcripts of these genes in the agro-infiltrated plants were quantified with qRT-PCR. Result showed that transcripts of *SlGC6*, *SlGC17* and *SlGC18* in the agro-infiltrated plants all dropped to lower than 30% of those in the eGFP control plants (Fig. 4C). This result demonstrated that the *SlGC* genes had been efficiently silenced in these plants. Taken together, silencing of *SlGC18* and co-silencing of *SlGC17/SlGC18* resulted in the TRV viral symptoms and higher level of virus accumulation. Thus, *SlGC17* and *SlGC18* redundantly and positively affect tomato resistance to TRV.

**Figure 5:**
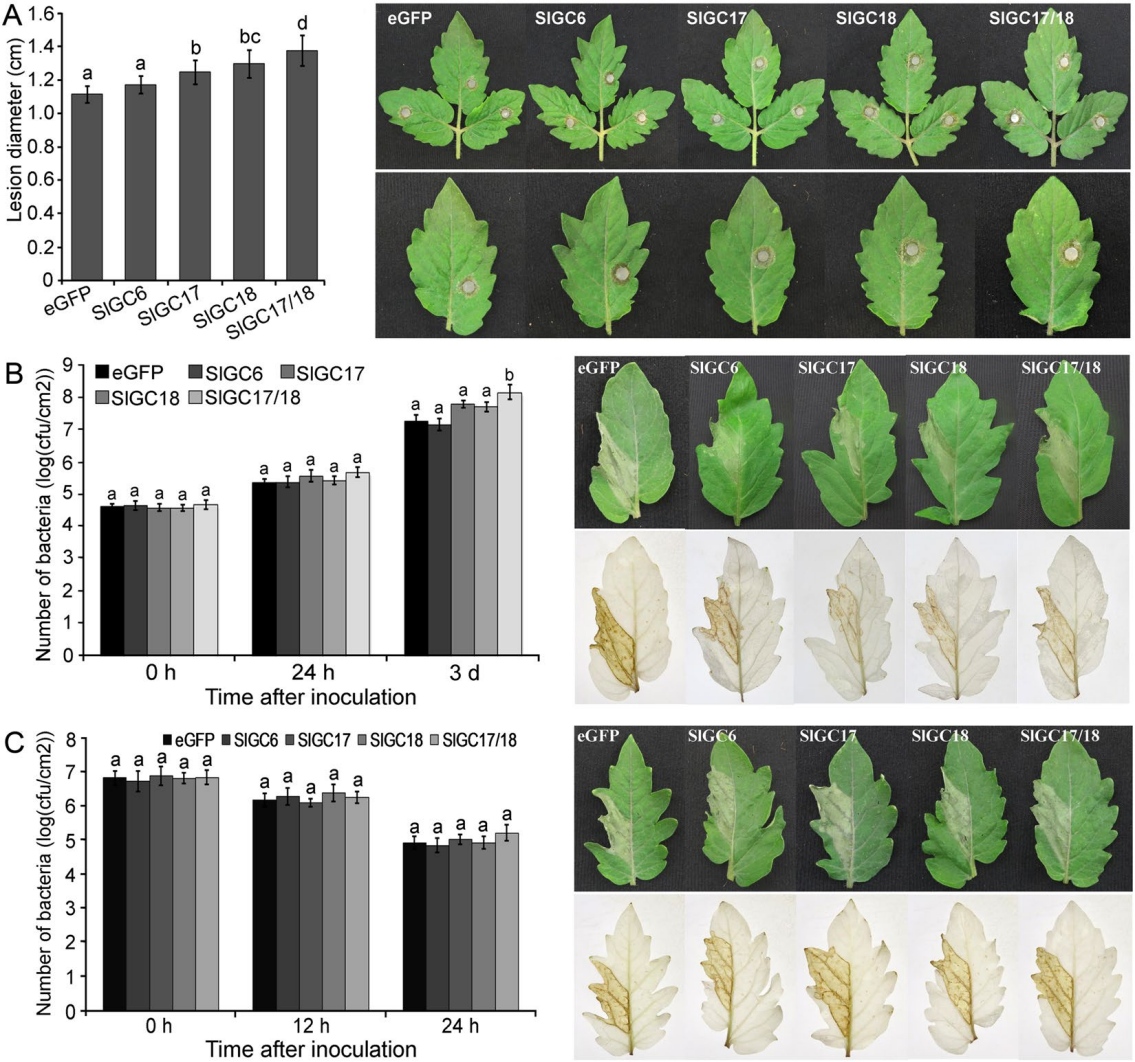
Silencing of *SlGC17* and *SlGC18* in tomato reduced resistance to *Sclerotinia sclerotiorum* (**A**) and *Pst* DC3000 (**B**) but not to *Xoo* (**C**). (**A**) *S. sclerotiorum* mycelial plug inoculation caused necrosis symptoms in silenced plants and statistical analysis of lesion diameter. Photographs were taken at 24 hpi. (**B**) *Pst* DC3000 cell suspension infiltration-inoculation caused disease symptoms in silenced plants and bacterial number counting assay of the inoculated areas. Photographs were taken at 3 dpi. (**C**) *Xoo* cell suspension infiltration-inoculation caused HR in silenced plants and bacterial number counting assay of the inoculated areas. Photographs were taken at 24 hpi. H_2_O_2_ in bacteria-inoculated leaves was detected by DAB staining (**B,C**). All above data represent mean ± SE of three independent experiments. Significant difference between the values for the silenced plants and those for the eGFP-control plants is indicated as small letters (*p* < 0.05, DMRT).

To further probe function of these three tomato GC genes in disease resistance, the silenced tomato plants were inoculated with three different pathogens including bacterial pathogen *Pseudomonus syringae* pv. *tomato* (*Pst*) DC3000, nonhost bacterial pathogen *Xanthomonus oryzae* pv. *oryzae* (*Xoo*) and a fungal pathogen *Sclerotiania sclerotiorum*. Resistance to all three pathogens in the *SlGC6*-silenced tomato plants appeared to be similar to that in the eGFP-control plants, as both plants developed similar severity of hypersensitive response (HR) or necrosis symptoms and accumulated similar levels of pathogens (Fig. 5). Nonhost resistance to *Xoo* in the *SlGC17* and *SlGC18* individually silenced and co-silenced plants was also similar to that in the eGFP control plants, judged by both HR symptoms and bacterial number counting results (Fig. 5C). These results indicated that the tomato BRI1-GC gene *SlGC6* might be not important in tomato resistance to the examined three pathogens and the putative tomato PEPR1-GC genes *SlGC17* and *SlGC18* might have no significant role in tomato nonhost resistance to *Xoo*.

However, when inoculated with *S. sclerotiorum*, necrotic symptoms of the leaves of both *SlGC17* and *SlGC18* indivudually silenced and co-silenced plants were significantly more severe than that of the eGFP control plants (Fig. 5A). The lesions in the *SlGC*-silenced plants, 1.2 ~ 1.4 cm at diameter, were significantly larger in size than those in control plants (1.1 cm at diameter) at 24 hpi (Fig. 5A). Interestingly, the lesion size was larger in co-silenced plants (1.35 cm at diameter) than that in individually silenced plants (<1.25 cm at diameter), indicating the redundancy of *SlGC17* and *SlGC18* in positively affecting tomato resistance to *S. sclerotiorum*.

Results of inoculation with *Pst* DC3000 showed that the necrotic disease symptoms of the leaves of *SlGC*-silenced plants were less severe than those in the eGFP control plants (Fig. 5B). Furthermore, *Pst* DC3000 bacterial growth in *SlGC17* and *SlGC18* co-silenced plants was signigicantly increased by approximately one order of magnitude, while that in *SlGC17* and *SlGC18* individually silenced plants was increased by about 0.6 order of magnitude, compared with that in the eGFP control plants at 3 d post inoculation (Fig. 5B). These data revealed that the *SlGC17* and *SlGC18* redundantly and positively regulate resistance to *Pst* DC3000 in tomato plants.

To examine whether H_2_O_2_ production was associated with *SlGC17* and *SlGC18*-mediated resistance to *Pst* DC3000, leaves of *SlGC*-silenced and eGFP control plants were stained *in situ* with 3,3-diamino benzidine hydrochloride (DAB) at 3 d post *Pst* DC3000 inoculation. The result showed that the leaves of *SlGC*-silenced plants were stained weaker than those of the eGFP control plants (Fig. 5B), manifesting that the former accumulated less H_2_O_2_ than the latter. This result indicated that oxidative burst appears to be associated with *SlGC17* and *SlGC18*-mediated resistance to *Pst* DC3000.

Collectively, the putative PEPR-GC genes *SlGC17* and *SlGC18* redundantly and positively regulate resistance to *S. Sclerotiorum* and *Pst* DC3000 in tomato plants. Furthermore, oxidative burst seems to participate in *SlGC17* and *SlGC18*-mediated resistance.

### Silencing of *SlGC17* and *SlGC18* in tomato reduced PAMP-triggered Ca^2+^ and ROS burst

**Figure 6:**
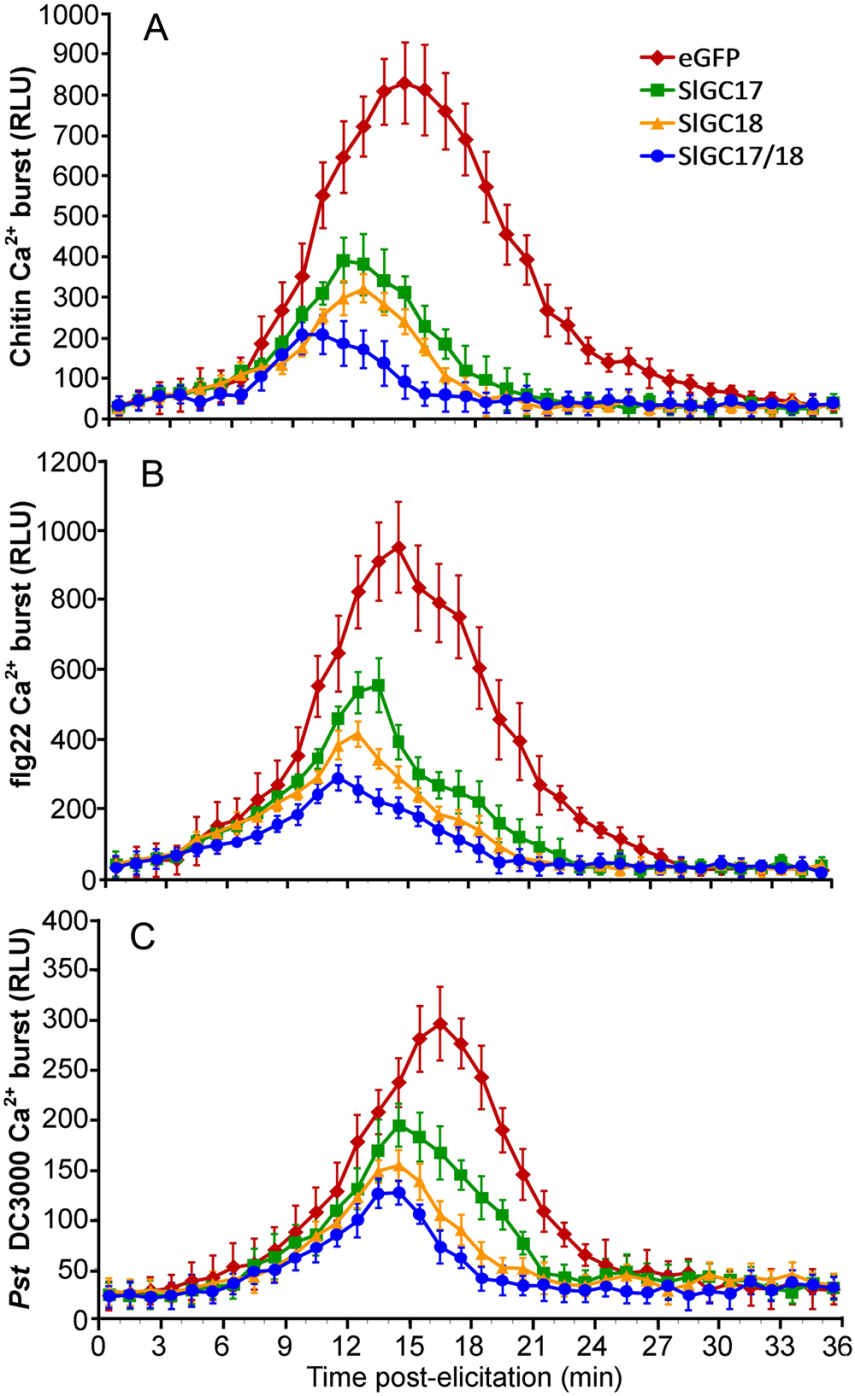
Silencing of *SlGC17* and *SlGC18* in tomato reduced PAMP-elicited and *Pst* DC3000 bacterial cells-induced Ca^2+^ burst. VIGS was performed in seeedlings of Aequorin-expressed transgenic tomato lines. Ca^2+^ induced by 100 nM flg22 (**A**), 100 μg mL^−1^ chitin (**B**) and *Pst* DC3000 bacterial cells (OD_600_ = 0.1) (**C**) in leaf discs of the silenced plants was measured as aequorin-based luminescence signal by using a Microplate Luminometer. Data represent mean ± SE of three independent experiments.

To further explore functions of *SlGC17* and *SlGC18* in pathogen-associated molecular pattern (PAMP)-triggered immunity (PTI) and the mechanisms underlying *SlGC17* and *SlGC18*-mediated resistance, effect of *SlGC17* and *SlGC18* gene silencing on the accumulation of Ca^2+^ and H_2_O_2_ elicited by fungal PAMP chitin, bacterial PAMP flg22 and *Pst* DC3000 bacterial cells was analyzed. Ca^2+^ burst was detected by an aequorin-based luminescence approach in aequorin-expressed transgenic (Aeq-OE) tomato plants. Both Ca^2+^ and H_2_O_2_ signals were measured and indicated as relative luminescence (RLU). In response to PAMP chitin, in eGFP control Aeq-OE plants, Ca^2+^ increased rapidly and peaked to 828 RLU at 12 min post chitin application, whereas the *SlGC17* and *SlGC18* individually or both silenced Aeq-OE plants although displayed similar dynamics of Ca^2+^ accumulation but exhibited significantly lower peak value with obvious difference (Fig. 6A). In the *SlGC17*- and *SlGC18*-silenced Aeq-OE plants, Ca^2+^ culminated to 392 and 322 RLU, respectively, while in the co-silenced Aeq-OE plants, the Ca^2+^ peak value was significantly reduced to 209 RLU, which was only one fourth of that in the control plants. These differences in Ca^2+^ burst was also observed for these plants in response to PAMP flg22, where Ca^2+^ peaked to 948 RLU in the eGFP control Aeq-OE plant, but culminated only to 289 RLU in *SlGC17/SlGC18* co-silenced Aeq-OE plants, and 553 and 414 RLU in *SlGC17*- and *SlGC18*-silenced Aeq-OE plants, respectively (Fig. 6B). In addition to the PAMPs, the response of these plants to *Pst* DC3000 bacterial cells was also monitored. In this case, Ca^2+^ peaked to 297 RLU in control Aeq-OE plants, 195 RLU in the *SlGC17*-silenced plants, 154 RLU in the *SlGC18*-silenced plants, and 127 RLU in the *SlGC17* and *SlGC18* co-silenced plants (Fig. 6C). These data demonstrated that although the response was weaker, the difference in Ca^2+^ burst elicited by *Pst* DC3000 bacterial cells was still clear between the *SlGC*-silenced plants and the eGFP control plants. Collectively, these results demonstrated the positive role of *SlGC17* and *SlGC18* in pathogen *Pst* DC3000-elicited and PAMP-triggered Ca^2+^ burst and the redundancy of these two genes in this role.

**Figure 7:**
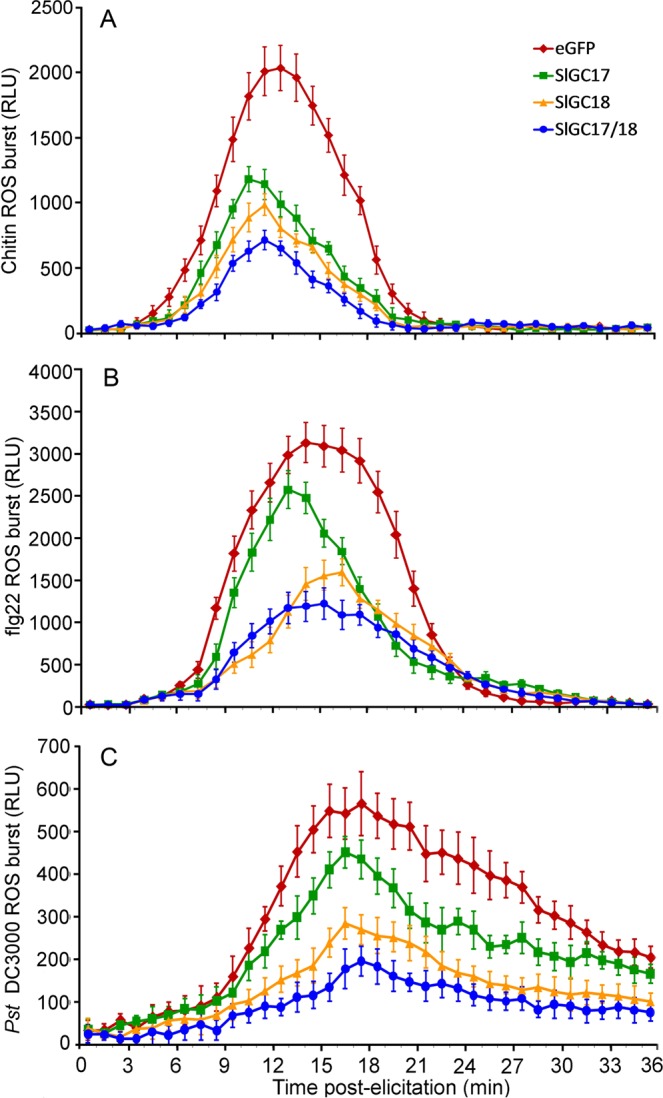
Silencing of *SlGC17* and *SlGC18* in tomato reduced PAMP-elicited and *Pst* DC3000 bacterial cells-induced ROS burst. H_2_O_2_ induced by 100 nM flg22 (**A**), 100 μg mL^−1^ chitin (**B**) and *Pst* DC3000 bacterial cells (OD_600_ = 0.1) (**C**) in leaf discs of the silenced plants was measured as luminol-based luminescence signal by using a Microplate Luminometer. Data represent mean ± SE of three independent experiments.

As shown in Fig. 7, the dynamics of H_2_O_2_ accumulation were similar to what were observed for Ca^2+^ measurement. Compared to the eGFP control plants, H_2_O_2_ accumulation was reduced most significantly in the *SlGC17* and *SlGC18* co-silenced plants, followed by in the *SlGC18*-silenced plants, and less significantly in the *SlGC17*-silenced plants, no matter in response to PAMPs chitin and flg22 or *Pst* DC3000 bacterial cells. The only difference was that the reduction of H_2_O_2_ accumulation caused by *SlGC17* silencing was less obvious in comparison to that of Ca^2+^ burst especially in response to flg22 and *Pst* DC3000 bacterial cells (Figs. 6 and 7).

Together, these results revealed that *SlGC17* and *SlGC18* redundantly play a positive role in pathogen *Pst* DC3000-elicited and PAMP-triggered Ca^2+^ and H_2_O_2_ burst.

### Silencing of *SlGC17* and *SlGC18* in tomato attenuated DAMP-triggered Ca^2+^ and ROS accumulation

**Figure 8:**
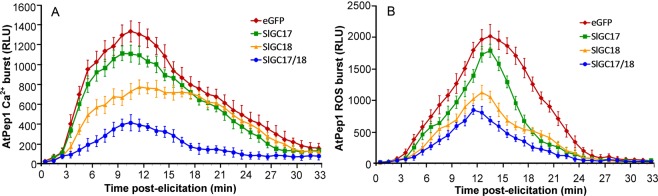
Silencing of *SlGC17* and *SlGC18* in tomato reduced DAMP-triggered Ca^2+^ and ROS burst. Detection of Ca^2+^ (**A**) and H_2_O_2_ (**B**) induced by 10 nM AtPep1 and the data processing followed what were described in Figs. 6 and 7.

AtPEPR1 recognizes AtPeps thereby activate plant defense responses including Ca^2+^ and H_2_O_2_ burst^22,23^. Therefore, function of *SlGC17* and *SlGC18* in DAMP-triggered Ca^2+^ and H_2_O_2_ burst in tomato was evaluated. In the eGFP control Aeq-OE tomato plants, Ca^2+^ increased rapidly and peaked to 1335 RLU at 10 min post AtPep1 application, while the *SlGC17* and *SlGC18* silenced Aeq-OE plants displayed similar dynamics of Ca^2+^ accumulation but showed lower peak value. The Ca^2+^ peak value dropped to 416 RLU in the *SlGC17* and *SlGC18* co-silenced Aeq-OE plants while it was less significantly lowered to 775 RLU in the *SlGC18*-silenced Aeq-OE plants. However, Ca^2+^ peak value (1115 RLU) in the *SlGC17*-silenced Aeq-OE plants was only slightly lower than that in the control plants (Fig. 8A). The difference of AtPep1-triggered H_2_O_2_ accumulation between the *SlGC*-silenced plants and the eGFP control plants was similarly observed for AtPep1-triggered Ca^2+^ burst. The peak value of H_2_O_2_ accumulation was 2016 RLU in the eGFP control plants, 1787 RLU in the *SlGC17*-silenced plants, 1125 RLU in the *SlGC18*-silenced plants, and 849 RLU in the *SlGC17* and *SlGC18* co-silenced plants (Fig. 8B). These results demonstrated that *SlGC17* and *SlGC18* redundantly play a positive role in DAMP-triggered Ca^2+^ and H_2_O_2_ burst and indicated that they are true functional homologs of AtPEPR1.

### Silencing of *SlGC17* and *SlGC18* in tomato altered expression of defense-related Ca^2+^ signaling genes

**Figure 9:**
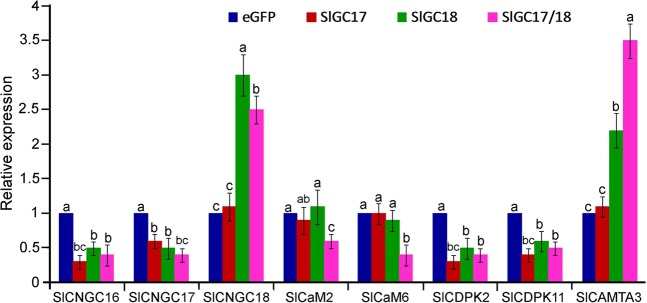
Silencing of *SlGC17* and *SlGC18* in tomato altered expression of a set of defense related Ca^2+^ signaling genes. Expression of *SlCNGC16*, *SlCNGC17*, *SlCNGC18*, *SlCaM2*, *SlaM6*, *SlCDPK2*, *SlCDPK11*, and *SlCAMTA3* genes in the *SlGC17* and *SlCNGC18* individually and together silenced plants as well as the eGFP non-silenced control plants was analyzed by qRT-PCR with gene-specific primers listed in Supplementary Table S1. Data represent mean ± SE of three independent experiments. Significant difference between the expression levels in the silenced plants and those in the eGFP control plants is indicated as small letters (*p* < 0.05, DMRT).

To further elucidate the molecular mechanisms underlying *SlGC*-mediated disease resistance, we monitored expression of a series of defense-related Ca^2+^ signaling genes to clarify whether they are involved in *SlGC-*mediated resistance. The checked genes under this expression analysis included three CNGC genes *SlCNGC16*, S*lCNGC17* and *SlCNGC18*, two calmodulin (CaM) genes *SlCaM2* and *SlCaM6*, two calcium-dependent protein kinase (CDPK) genes S*lCDPK2* and *SlCDPK11*, which are tomato homologs of *AtCPK2* and *AtCPK11*, and one CaM-binding transcription activator (CAMTA) gene *SlCAMTA3*. All these genes play an important role in regulating disease resistance^24,59–64^. The expression result showed that silencing of *SlGC17* and *SlGC18* individually or together unanimously reduced the expression of *SlCNGC16*, *SlCNGC17*, *SlCDPK2*, and *SlCDPK11* but only co-silencing of *SlGC17/18* lowered the expression of *SlCaM2* and *SlCaM6* by 40% and 60%, respectively. In addition, silencing of *SlGC18* and co-silencing of *SlGC17/SlGC18* increased the expression of *SlCNGC18* by 3.0 and 2.5 folds and that of *SlCAMTA3* by 2.2 and 3.5 folds, respectively (Fig. 9). Both *SlCNGC18* and *CAMTA3* have been found to be negative regulators of plant resistance to pathogens including *S. Sclerotiorum*^24,60,63,64^. These results indicated that CNGCs, CaMs, CDPKs, and CAMTAs may play a role in the *SlGC17* and *SlGC18-*mediated resistance.

### Cytoplasmic domain of SlGC17 and SlGC18 proteins exhibited *in vitro* GC activity

**Figure 10:**
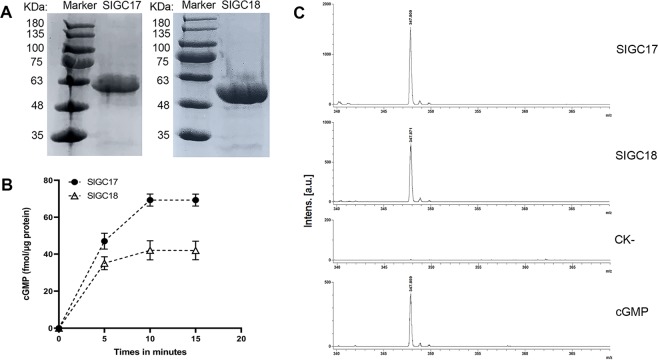
Cytoplasmic domain of SlGC17 and SlGC18 proteins exhibited *in vitro* GC activity. (**A**) SDS-PAGE profile of the affinity purified His-tagged intracellular domain of SlGC17 and SlGC18 proteins (SlGC17_724-1105_ and SlGC18_788-1104_). (**B**) Cyclic GMP generated after incubation of 10 µg of SlGC17_724-1105_ and SlGC18_788-1104_ proteins in 100 μl of reaction mixture containing 50 mM Tris-HCl (pH 8.0), 1 mM GTP, 2 mM IBMX and 5 mM MgCl_2_. The cGMP was determined by enzyme immunoassay based on highly specific anti-cGMP antibody. The error bars represent the standard error of the mean (n = 3). (**C**) MALDI-TOF-MS spectra of cGMP in the reaction mixture containing SlGC protein (CK-: no SlGC protein was added) and the substrate GTP as described in (**B**) after incubation for 10 min. The spectrum of standard cGMP was also shown at the bottom. The experiment was performed three times with similar results.

To examine whether the GC-CC-containing SlGC17 and SlGC18 proteins indeed possess GC activity as predicted by bioinformatics analyses, the intracellular domain of SlGC17 and SlGC18 proteins (SlGC17_724-1105_ and SlGC18_788-1104_) was subjected to assays for activity to generate cGMP from GTP. The cDNA sequences corresponding to SlGC17_724-1105_ and SlGC18_788-1104_ were amplified from tomato cv. Moneymaker, and His-tagged SlGC17_724-1105_ and SlGC18_788-1104_ were expressed in *E. coli* and affinity purified using anti-His antibody, showing a single band in SDS-PAGE gel with an expected molecular mass of approximate 62 kDa and 55 kDa, respectively (Fig. 10A). These purified proteins were then examined for their GC activity.

Results of enzyme immunoassay using highly specific anti-cGMP antibody showed that both SlGC17_724–1105_ and SlGC18_788–1104_ exhibited activity to produce cGMP from GTP (Fig. 10B). The cGMP was generated rapidly within 5 min after incubation of SlGC protein and GTP, and reached the maximum value at 10 min after incubation. The peak value of cGMP concentration catalyzed by SlGC17_724–1105_ is approximately 70 fmol/ µg protein, which was 1.8 times as high as that by SlGC18_788–1104_ (Fig. 10B), suggesting that SlGC17 owns higher catalysis activity than SlGC18.

To validate the results of enzyme immunoassay, cGMP produced from the reactions was identified by more sensitive MALDI-TOF-MS assay. Results of the MS assays again demonstrated that cGMP indeed generated in the incubation solution containing SlGC17_724–1105_ or SlGC18_788–1104_ and GTP (shown as a MS peak of about 347 m/z as for cGMP standard), but not in that without SlGC protein (CK-) (Fig. 10C), and thus confirmed the results of enzyme immunoassay.

Collectively, these results of *in vitro* assays revealed that cytoplasmic domain of SlGC17 and SlGC18 proteins exhibits GC activity.

## Discussion

cGMP has been recognized as an important second messenger in plants. However, to how wide range GCs exist and how they function in plants remain poorly understood. As a matter of fact, the majority of studies regarding plant GCs were conducted only in the model plant species Arabidopsis. In this study, we identified GC candidates in tomato genome and explored functions of two PEPR-GC genes in tomato resistance, representing the first genome-wide GC identification and functional study of PEPR-GC candidate genes in a crop plant species.

### GC family in tomato

Due to the lack of higher plant sequence with high similarity to known GCs in animals and fungi, no significant progress on identification of plant GCs has been made until motif BLAST search approach was employed. The motif is generated based on the conserved AA residues required for GC catalysis function, including those form the hydrogen bond with the guanine, confer substrate specificity for GTP, stabilize the transition state from GTP to cGMP, and interact with the Mg^2+^/Mn^2+^ ions^35,37,38^. Through using this motif BLAST search approach, seven *Arabidopsis thaliana* proteins that exhibit GC activity *in vitro* have been identified to date^22,38–43^. In addition, further motif searching study predicted 41 potential GCs in *A. thaliana* genome^35^. However, genome-wide identification of GCs in other higher plant species has not yet been conducted. In this study, employing phi-BLAST of the plant specific GC motif, we identified 99 candidate GCs in genome of the economically important crop plant tomato (Fig. 1, Supplementary Table S3), reperesenting the largest number of GCs predicted in a plant species. Together with the prediction in Arabidopsis^35^, our work revealed that a large GC family is present in a plant species and it is expected that GCs widely exist in higher plants.

Notably, all the 99 tomato GC candidates identified in this study are kinases and the GC-CC is embedded in the kinase domain (Fig. 2). Our finding is consistent with what was obtained in the previous study in Arabidopsis^35,37^. Collectively, these results demonstrate that GC-CC is frequently encapsulated in a kinase domain. Here, we name these GC-CC-containing kinases as GC-kinases. They appear to exist widely in plants. Nevertheless, it needs to be pointed out that only a portion of kinase repertoire in a plant species belong to GC-kinases. For example, tomato genome contains over 640 receptor-like kinases (RLKs)^65^. But we found that only about 100 tomato kinases are GC-kinases. Moreover, whether a kinase embeds a GC-CC motif seems not to correlate with the type of kinase, since diverse types of kinases belong to GC-kinases. Tomato GC-kinases include LRR-Pkinase/Pkinase_Tyr, CB_EGF-Pkinase, MB_Lectin-Pkinase_Tyr/Pkinase, SSR/AF-Pkinase/Pkinase_Tyr, and Pkinase or Pkinase_Tyr type kinases possessing no any other known domain (Fig. 2, Supplementary Table S3). Further, not all members of the same type of kinases in a plant species belong to GC-kinases. For instance, only six members of AtWAKL family and seven members of AtCRK family were predicted to be GC-kinases (Fig. 3, and ref. ^35^).

Additionally, alignment of the GC-CC motif region of the 99 tomato GCs revealed a tomato GC-CC motif as ([SK] [FYA] [GS] [VNIY] [VIL] [LVFMID] [LAMV] [EDM] [LITAE] [LIVEDA] [TSMLIC] [GNSRDIC] [RKPQLM] [RK] x{0,2} [DSEG]) (Fig. 1). Comparison of this motif with the one previously reported for plant ([KS] [YF] [GCS] [VIL] [VILFG] [DVIL] [VILADG] [EPVIL] [DVIL] [TVIL] [WST] [PDRG] [KEG] [KR] x{2,3} [DHSE])^35^ demonstrated that the tomato GC-CC motif is more relaxed at some positions not directly involved in catalysis function. For example, A2, N4, D8, T9, [ED]10, M11, [GN]12, [RPQL]13 appearred frequently in predicted tomato GCs but not included in the plant GC-CC motif. These amino acids except [G]12 and [RQL]13 present mainly in but not strictly limited to SlGC90-SlGC99, which belong to S-locus lectin protein kinase family and clustered with AtB120 in the same clade of group VII (Fig. 3, Supplementary Table S3), while [G]12 and [R]13 are the amino acids most frequently present at these positions. In addition, many amino acids present in only one SlGC also do not appear in the plant GC-CC motif. These amino acids include [DVNTP]1, [ACL]4, [NQP] 5, [CAW]6, [PT]7, [S]8, [FKNYP]9, [CEMN]10, [FPYN]11, [LHTA]12 and [AIH]13. Collectively, our result suggests that the plant GC-CC motif needs to be relaxed for these positions when used for phi-BLAST search for plant GC prediction.

### Role of SlPEPR-GCs in disease resistance

In the present study, we focused on the role of GC-CC-containing tomato homologs of AtPEPR (SlPEPR-GC) in disease resistance. AtPEPR1 is a LRR-RLK that recognizes Pep elicitors and triggers immunity signaling in Arabidopsis. It contributes to resistance against the bacterial pathogen *Pst* DC3000^23^. Here, we obtained similar results for the two SlPEPR-GCs (SlGC17 and SlGC18). Silencing of *SlGC17* and *SlGC18* reduced tomato resistance to this pathogen (Fig. 5B). Moreover, we demonstrated that *SlGC17* and *SlGC18* play a positive role in tomato resistance to other types of pathogens including fungal pathogen *S. sclerotiorum* and viral pathogen TRV (Figs. 4 and 5). In addition, SlGC17 and SlGC18 are also LRR-RLK proteins (Supplementary Table S3) and more importantly play a positive role in AtPep1-triggered Ca^2+^ and H_2_O_2_ burst (Fig. 8). Together, our results indicate that SlGC17 and SlGC18 are most probably the functional orthologs of AtPEPR and play important roles in disease resistance against diverse pathogens.

*SlGC17* and *SlGC18* exhibited identical direction but different level in all functions examined in this study including affecting resistance to various pathogens, PAMP and DAMP triggered Ca^2+^ and H_2_O_2_ burst as well as Ca^2+^ signaling gene expression. *SlGC18* displayed stronger regulatory role in comparison with *SlGC17*, while *SlGC17* and *SlGC18* together showed more substantial effects in all these functions compared with single gene (Figs. 4–9). These results revealed that *SlGC17* and *SlGC18* function redundantly but *SlGC18* acts more strongly than *SlGC17*. The overlapped functions have been observed for AtPEPRs^23^.

It has been reported that AtPEPR1 has GC activity, generating cGMP from GTP, and that cGMP can activate AtCNGC2-dependent cytosolic Ca^2+^ elevation and act in pathogen defense signaling cascades in Arabidopsis^22,23^. Our evidences support SlGC17 and SlGC18 to be GCs. First, SlGC17 and SlGC18 both contain a GC-CC motif which carries all AA residues required for GC catalysis activity (Fig. 1). Second, silencing of *SlGC17* and *SlGC18* significantly reduced Ca^2+^ burst evoked by PAMPs (flg22 and chitin), DAMP (AtPep1) and *Pst* DC3000 living bacterial cells (Figs. 6 and 8), demonstrating the important positive role of *SlGC17* and *SlGC18* in these Ca^2+^ burst responses. Moreover, cGMP and tomato homologs of *AtCNGC2* contribute to resistance in tomato against various pathogens including *S. sclerotiorum*^24,58,62^. In addition, our previous studies reveal that various Ca^2+^ signaling related genes including some *CaMs*, *CDPKs* and related genes as well as *CAMTA3* are involved in various types of resistance in different plant species including tomato^56,59,63,64,66,67^, and silencing of *SlGC17* and *SlGC18* altered the expression of a set of *CNGC*, *CDPK*, *CaM* and *CAMTA3* genes (Fig. 9). Finally and more importantly, enzyme immunoassay and MALDI-TOF-MS assays revealed that the GC-CC-containing cytoplasmic domain of SlGC17 and SlGC18 exhibits *in vitro* GC activity (Fig. 10).

The elucidation of functional mechanism of GC-kinases is complicated by the finding that the GC-CC is embedded in the kinase domain. It will be intriguing to clarify the relationship of GC-CC and kinase by analyzing the mutual effects on each other in regulating biological processes such as disease resistance. Do they separately contribute to the functions of GC-kinases, or coordinate in the regulating functions? The currently available examples suggest that this might be GC-kinase-dependent, AtPSKR1 seemed to support separation model while AtBRI1 appearred to favor coordination model^45,68^. Muleya *et al*.^68^ reported that calcium acted as the switch in the moonlighting dual function of the ligand-activated receptor kinase AtPSKR1. However, in case of AtBRI1, a functional kinase was required for GC activity to generate cGMP, which rapidly potentiates phosphorylation of the downstream substrate BSK1^45^. Additionally, considering that kinase exists in a plant species as a superfamily with large number of members, the embedding of a GC-CC in a kinase domain should efficiently broaden the range of processes for GC regulation. This may also at one layer account for the wide range of GC/cGMP-regulated biological processes.

**Figure 11:**
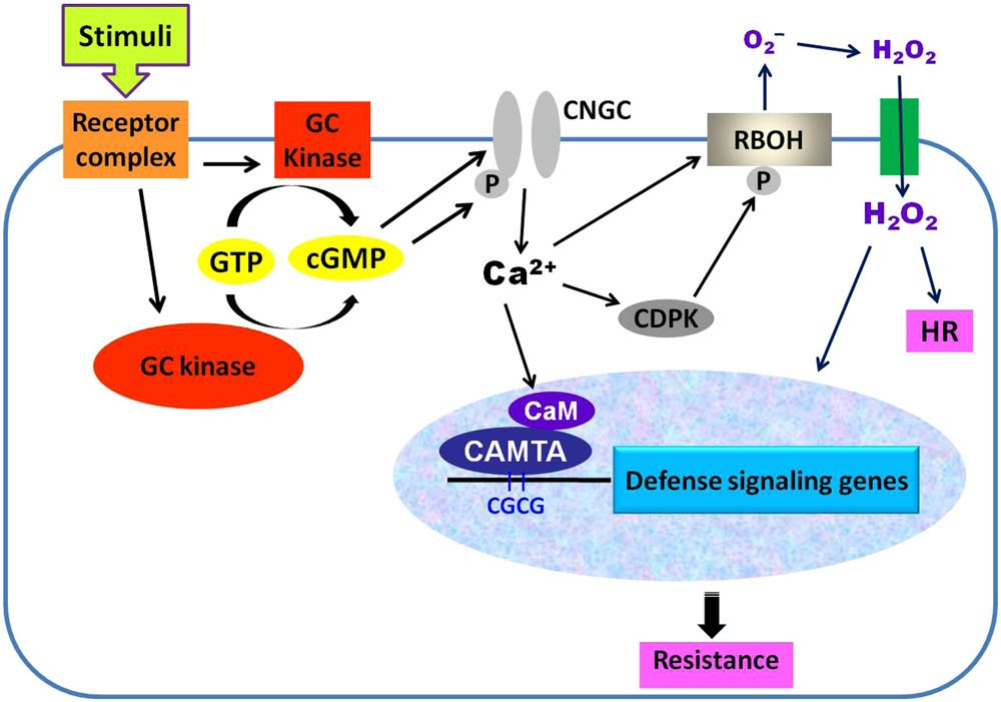
A hypothetical model for cGMP signal transduction in plant resistance. Pathogen-related stimuli are recognized by plant receptor proteins. This recognition leads to activation of cytoplasmic or transmembrane GC-kinases and consequently an accumulation of cGMP, which causes cytosolic Ca^2+^ influx through opening of CNGC channels. The cytosolic Ca^2+^ modulates CDPK-mediated and RBOH-dependent ROS accumulation as well as CAMTA3-mediated denfense signaling, thereby regulates HR and disease resistance. Abbreviations: CaM, calmodulin; CAMTA, calmodulin binding transcriptional activator; CDPK, calcium-dependent protein kinase; CNGC, cyclic nucleotide gated channel; GTP, guanosine triphosphate; cGMP, cyclic guanosine monophosphate; GC, guanylate cyclase; HR, hypersensitive response.

Based on our findings, we propose a working model for cGMP signal transduction in the resistance of tomato (Fig. 11). In this model, stimuli including pathogens such as *S. sclerotiorum*, and *Pst* DC3000 as well as PAMPs such as chitin and flg22 and DAMPs such as AtPep1 activate GCs embedded within kinases of transmembrane RLKs or cytoplasmic RLCKs to generate cGMP, which activates Ca^2+^ channels such as CNGCs through direct binding to them or promoting their phosphorylation, leading to cytosolic Ca^2+^ influx. The cytosolic Ca^2+^ signal is transduced by various Ca^2+^ sensor proteins including CaM, which regulates CAMTA3. CAMTA3 directly binds to the CGCG *cis*-elements in the promoter of defense-related target genes and regulates their expression, which alters plant disease resistance. Simultaneously, the increased cytosolic Ca^2+^ also enhances RBOH activity by direct binding or CDPK-mediated phosphorylation, resulting in ROS accumulation and thereby affecting hypersensitive response (HR) and plant disease resistance (Fig. 11).

## Conclusions

Ninety-nine candidate GC-kinases, which embedded a GC catalytic center (GC-CC) motif within the protein kinase domain, were identified in tomato genome. Two homologs of Arabidopsis PEPRs, SlGC17 and SlGC18 exhibited *in vitro* GC activity. Co-silencing of *SlGC17* and *SlGC18* genes significantly reduced resistance to a variety of pathgens including *Sclerotinia sclerotiorum* and *Pseudomonas syringae* pv. *tomato* (*Pst*) DC3000, and attenuated PAMP- and DAMP-elicited Ca^2+^ and H_2_O_2_ burst. Additionally, silencing of these genes altered the expression of a set of Ca^2+^ signaling genes including *SlCNGCs*, *SlCaMs*, *SlCDPKs* and *SlCAMTA3*. Co-silencing of *SlGC17* and *SlGC18* caused stronger effects than individual silencing. Collectively, our results reveal that GC-kinases widely exist in tomato and the two *SlPEPR-GC* genes redundantly play a positive role in resistance to diverse pathogens and PAMP/DAMP-triggered immunity in tomato.

## Supplementary information

Supplementary material.
