# Supplementary material for: Characterization of tomato protein kinases embedding guanylate cyclase catalytic center motif
Source: Sci Rep. 2020 Mar 5;10:4078. doi: 10.1038/s41598-020-61000-7 (PMC7057975; doi:10.1038/s41598-020-61000-7)
Supplement: Supplementary file 1 — Supplementary material. [file 41598_2020_61000_MOESM1_ESM.pdf]

Supplementary information

Characterization of tomato protein kinases embedding guanylate cyclase catalytic center motif

Hafizur Rahman[1a], Xin-Yao Wang[1a], You-Ping Xu[2], Yu-Han He[1], and Xin-Zhong Cai[1*]

Table S1 Primers used in this study

Target Gene	Primer Name	Sequence (5'-3')
For VIGS constructs		
SlGC6	SlGC6 F	aggatccCAGCTTAGTACACTTCCTGC
	SlGC6 R	agaattcTCAATCTTCCATGCTGAAGC
SlGC17	SlGC17 F	aggatccAACCAGTGCTCCGCCGACAA
	SlGC17 R	agaattcGGTGGGCCAAGTTCAACGT
SlGC18	SlGC18 F	aggatccTGATGGAATGCATTCATTGTT
	SlGC18 R	cgaattcAAGCAATTACTAGGAGTAAGG
For qRT-PCR expression analyses		
SlGC6	qSlGC6 F	GGAAATCAATGGAAGTGATTG
	qSlGC6 R	TTGATGCAAAATCCTCTACAC
SlGC17	qSlGC17 F	GGAATGATGAAGCAGAAGTTT
	qSlGC17 R	CAGGCATAAGTTCTCTCAACA
SlGC18	qSlGC18 F	GCTCTTAGATGTACAGAGAA
	qSlGC18 R	GAGCTAGAAGTGAGACATCA
TRV2 2b	TRV2 2b-F	ATGCACGAATTACTTAGGAAG
	TRV2 2b-R	GGTAACCTTACTCACAGAAT
TRV1	TRV1 Rep-F	ATCTCAAGTTGATTTGAGGTT
	TRV1 Rep-R	TGATCTCTTTGCTTACATCGT
SlCaM2	SlCaM2-F	CTGATGAAGAAGTCGATGAGATG
	SlCaM2-R	AGACAAGAGCCTACCCAATGA
SlCaM6	SlCaM6-F	ATGAGATGATCCGAGAGG
	SlCaM6-R	AGGCCACTAATATACTTGAACC
SlCNGC16	SlCNGC16-F	ACCTGATAACAGCGACGACA
	SlCNGC16-R	TGAATTGGAACTAGAGAAGA
SlCNGC17	SlCNGC17-F	CTGAAGATGAAGATGAAGAT
	SlCNGC17-R	GTTTCTGTCATCACGACTA
SlCNGC18	SlCNGC18-F	TTATAGTGATCGAAGTGACG
	SlCNGC18-R	AAGAAGTCTGTCAATCCTCC
SlCDPK2	SlCDPK2-F	ATAAGATATTGAACCGTTGG
	SlCDPK2-R	GTCATCCGGCACCCGAGCC
SlCDPK11	SlCDPK11-F	CTACTCTCAGCAACAGCATG
	SlCDPK11-R	TGAACTAGAGGAAAAACCCT
SlCAMTA3	SlCAMTA3-F	TGATGCATGAACAAGGAAAC
	SlCAMTA3-R	TCAACTTCTGATCCATGGAC
SlrRNA	SlrRNA-F	GCCGGCGACGCATCATTCAAA
	SlrRNA-R	CGCGCCTGCTGCCTTCCTT

Table S2 GC-CC motifs of SlGCs predicted using GCPred tool

Hit-No	Position	GCC-sequence	GCC-Hydrophobicity	GCC-Molecular-Weight	GCC-Isoelectric-point	GCC-Mean
SlGC1						
1	608-623	SNSLSGQIPADLGRLS	0.541	0.768	0.901	0.737
2	850-865	KACYNDGMVLSIRRLS	0.646	0.72	0.85	0.739
3	608-624	SNSLSGQIPADLGRLSH	0.541	0.768	0.901	0.737
SlGC2						
1	865-879	SDGMLLSICRLPDRS	0.667	0.851	0.802	0.773
2	1032-1046	SFGIVLLELLTGKKS	0.786	0.85	0.872	0.836
3	619-634	SNSLSGQIPSDLARLS	0.49	0.795	0.901	0.729
4	619-635	SNSLSGQIPSDLARLSH	0.49	0.795	0.901	0.729
5	861-877	KACYSDGMLLSICRLPD	0.606	0.762	0.906	0.758
6	865-881	SDGMLLSICRLPDRSLD	0.667	0.851	0.802	0.773
7	392-408	KCSSLSVLDLEGNRFIG	0.656	0.841	0.877	0.791
SlGC3						
1	1091-1106	KVGLLCTMPDPLERPS	0.651	0.841	0.852	0.781
SlGC4						
1	149-164	SCSYLNKLMLNDNKLS	0.713	0.82	0.831	0.788
2	253-268	KAGKRRKMGYGLGRVD	0.606	0.567	0.704	0.626
3	253-269	KAGKRRKMGYGLGRVDS	0.606	0.567	0.704	0.626
4	488-504	SFGVVLLELATGQKPLE	0.778	0.82	0.916	0.838
5	253-269	KAGKRRKMGYGLGRVDS	0.606	0.567	0.704	0.626
SlGC5						
1	485-501	SFGVVLLELATGQKPLE	0.778	0.82	0.916	0.838
SlGC6						
1	961-976	KSSNVLLDNEMDARVS	0.564	0.849	0.826	0.747
2	961-977	KSSNVLLDNEMDARVSD	0.564	0.849	0.826	0.747
3	1021-1037	SFGVVLLELLTGKRPTD	0.783	0.846	0.872	0.834
SlGC7						
1	658-673	KTGADSWKMTAFQKVE	0.616	0.67	0.842	0.709
2	848-863	SYGYIAPEYAYTLRVD	0.699	0.747	0.913	0.786
3	51-67	SVCSWVGIKCLQDRVVS	0.701	0.765	0.821	0.763
4	848-864	SYGYIAPEYAYTLRVDE	0.699	0.747	0.913	0.786
5	658-674	KTGADSWKMTAFQKVEF	0.616	0.67	0.842	0.709
6	870-886	SFGVVLLELITGRRPVG	0.782	0.822	0.855	0.82
SlGC8						
1	63-77	SICSWTGITCDDTKS	0.683	0.805	0.835	0.774
2	842-857	SYGYIAPEYAYTLKID	0.699	0.747	0.913	0.786
3	842-858	SYGYIAPEYAYTLKIDE	0.699	0.747	0.913	0.786
4	63-79	SICSWTGITCDDTKSVT	0.683	0.805	0.835	0.774
SlGC9						
1	53-67	SHCTWNGVTCDTHRH	0.648	0.81	0.861	0.773
2	270-284	SLSGSLTPEIGYLKS	0.569	0.732	0.913	0.738
3	856-871	SYGYIAPEYAYTLKVD	0.699	0.747	0.913	0.786
4	856-872	SYGYIAPEYAYTLKVDE	0.699	0.747	0.913	0.786
5	53-69	SHCTWNGVTCDTHRHVT	0.648	0.81	0.861	0.773
6	270-286	SLSGSLTPEIGYLKSLK	0.569	0.732	0.913	0.738
7	878-894	SFGVVLLELVSGKKPVG	0.78	0.83	0.874	0.828

SlGC10						
1	859-874	SYGYIAPEYAYTLKVD	0.699	0.747	0.913	0.786
2	714-730	SNGIDVAIKKLVGRGTG	0.584	0.829	0.795	0.736
3	881-897	SFGVVLLELITGHKPVG	0.769	0.838	0.906	0.838
SlGC11						
1	876-891	SYGYIAPEYGYTLKVD	0.675	0.734	0.913	0.774
2	876-892	SYGYIAPEYGYTLKVDE	0.675	0.734	0.913	0.774
3	898-914	SFGVVLMELLTGKRPLD	0.8	0.832	0.876	0.836
4	791-807	SLGEVLHGKQAAGRLLV	0.697	0.692	0.828	0.739
SlGC12						
1	863-877	SYGVVLLEILSGKRS	0.767	0.831	0.873	0.824
2	806-822	KPSNILLDGDLEARVAD	0.53	0.805	0.805	0.713
3	863-879	SYGVVLLEILSGKRSVE	0.767	0.831	0.873	0.824
SlGC13						
1	901-915	SYGVVLMEILSGKRS	0.784	0.817	0.877	0.826
2	494-510	SYSGLIGNLPDFKRCQS	0.582	0.826	0.846	0.751
3	844-860	KPSNILLDGEMEARVAD	0.562	0.798	0.815	0.725
4	901-917	SYGVVLMEILSGKRSVD	0.784	0.817	0.877	0.826
SlGC14						
1	22-36	SSSPRDIAILLRVKS	0.567	0.769	0.782	0.706
2	79-94	SFGIAGRFPADFCRIS	0.578	0.734	0.818	0.71
3	211-227	SIGRLGKLRIFYARFAS	0.601	0.687	0.752	0.68
4	854-870	SYGYIAPEYAYTLKITE	0.699	0.747	0.913	0.786
5	876-892	SFGVVLLELIIGKRPND	0.748	0.859	0.87	0.826
SlGC15						
1	389-403	SFSGNIPASLGNCRS	0.672	0.791	0.912	0.791
2	397-411	SLGNCRSLLRIRFRS	0.589	0.768	0.722	0.693
3	879-895	SFGVVILELVTGKRPVS	0.793	0.845	0.872	0.837
4	461-477	KFSGVIPSEVGKLKNLV	0.658	0.791	0.881	0.777
5	656-672	KKGNTMTKWTSFHKLGF	0.592	0.748	0.817	0.719
6	701-717	SNGEAVAVKKLWERTVK	0.59	0.739	0.753	0.694
SlGC16						
1	452-467	SASNLSALILSKNKFS	0.739	0.808	0.882	0.81
2	695-710	SGSSGKVYKVVLSKGD	0.613	0.723	0.84	0.725
3	864-880	SCGYIAPEYAYTLRVNE	0.691	0.715	0.907	0.771
4	886-902	SFGVVILELVTGKRPVD	0.793	0.845	0.872	0.837
5	452-468	SASNLSALILSKNKFSG	0.739	0.808	0.882	0.81
SlGC17						
1	221-237	SISGFLPSSLGQLKRLE	0.645	0.733	0.931	0.77
2	804-820	SMSAFSSEIATLARIRH	0.661	0.771	0.924	0.785
3	932-948	SYGYFAPEYACMLKITE	0.675	0.717	0.898	0.764
4	954-970	SFGVVLLEIITGKKPAD	0.78	0.846	0.871	0.833
SlGC18						
1	1005-1021	SYGIVLLELITRKKVLD	0.793	0.85	0.808	0.817
SlGC19						
1	351-367	KSSNILLDEFCKPRIAD	0.641	0.814	0.836	0.764
2	411-427	SFGVVLMELISGKRPIE	0.784	0.817	0.876	0.826
SlGC20						
1	57-71	SKSPCKFYGIQCDKH	0.602	0.789	0.77	0.72
2	322-337	SNSFTGEIPANLGRFS	0.556	0.747	0.907	0.737
3	540-555	KLSGSIPPSLDNLKLS	0.55	0.836	0.881	0.756

4	540-556	KLSGSIPPSLDNLKLSS	0.55	0.836	0.881	0.756
5	704-720	KKGCGTVAVKQLWKGNE	0.589	0.772	0.823	0.728
6	872-888	SFGVVLLELVTGRKPIE	0.795	0.821	0.856	0.824
7	29-45	SLSVETEALLEFKKQLV	0.675	0.798	0.791	0.754
SlGC21						
1	84-98	SLSGVISPSIFSLKS	0.629	0.819	0.936	0.795
2	869-885	SFGVVLLELVTGRKPIE	0.795	0.821	0.856	0.824
3	84-100	SLSGVISPSIFSLKSLT	0.629	0.819	0.936	0.795
SlGC22						
1	843-859	SYGIVLLELLTGRKAVD	0.786	0.826	0.856	0.823
SlGC23						
1	837-853	SFGIVLLELLTGKKPVD	0.786	0.85	0.872	0.836
SlGC24						
1	945-959	KNCRDLVSWVFQLKS	0.572	0.761	0.829	0.721
2	242-257	KLSKLKSLVLSGNRFH	0.712	0.811	0.834	0.786
3	335-350	SLSSRELKILSLAKNE	0.573	0.772	0.803	0.716
4	945-960	KNCRDLVSWVFQLKSE	0.572	0.761	0.829	0.721
5	924-940	SFGVVLLELLTGKRPVE	0.783	0.846	0.872	0.834
SlGC25						
1	212-227	KLSRLTVLSLQENRFS	0.737	0.845	0.822	0.801
2	871-887	SFGVVLLELLTCKRPMD	0.756	0.887	0.857	0.833
SlGC26						
1	163-178	KSSNILLGHNMEARVS	0.632	0.783	0.868	0.761
2	222-237	SFGVVLLELLTGKKPS	0.783	0.846	0.872	0.834
3	163-179	KSSNILLGHNMEARVSD	0.632	0.783	0.868	0.761
4	222-238	SFGVVLLELLTGKKPSD	0.783	0.846	0.872	0.834
SlGC27						
1	849-864	SNCIGSGGFGATYKAE	0.651	0.629	0.947	0.742
2	241-257	SGSIPGEIGRSCEKLQS	0.585	0.679	0.783	0.683
3	1031-1047	SYGVVLLELISDKKALD	0.769	0.868	0.82	0.819
4	667-683	SLSGEIPNNLVNLRNLT	0.573	0.842	0.899	0.772
SlGC28						
1	259-274	KCSHLRTVLLNSNKFS	0.724	0.825	0.841	0.797
2	457-472	SYCIGTGGFGSTYRVE	0.683	0.683	0.949	0.772
3	272-288	KFSGVIPSELGGLRKLE	0.634	0.768	0.927	0.776
4	638-654	SYGVVLLELLSDKRALD	0.77	0.868	0.821	0.819
5	457-473	SYCIGTGGFGSTYRVEI	0.683	0.683	0.949	0.772
SlGC29						
1	177-191	SSSTQSGDKYQIHKS	0.551	0.763	0.853	0.722
2	177-193	SSSTQSGDKYQIHKSLS	0.551	0.763	0.853	0.722
3	435-451	SYGVVLLELITGKRAIE	0.782	0.846	0.872	0.833
4	172-188	SLSVSSSSTQSGDKYQI	0.691	0.774	0.901	0.789
SlGC30						
1	452-468	KSSNILLDENFVAKVAD	0.487	0.833	0.885	0.735
2	514-530	SYGVVLLELITGRRAIQ	0.782	0.822	0.855	0.82
SlGC31						
1	50-64	SCSLNSWYVIKCDRS	0.686	0.776	0.823	0.761
2	50-65	SCSLNSWYVIKCDRSS	0.686	0.776	0.823	0.761
3	506-521	KSSNILLDEKYRAKVS	0.569	0.765	0.775	0.703
4	50-66	SCSLNSWYVIKCDRSSD	0.686	0.776	0.823	0.761
5	506-522	KSSNILLDEKYRAKVSD	0.569	0.765	0.775	0.703

6	565-581	SFGVVLAELLTGKKAIS	0.821	0.842	0.872	0.845
SlGC32						
1	300-315	KDGCQDIDECIDRKPD	0.542	0.837	0.695	0.691
SlGC33						
1	108-123	SHCIDSDTGLGGYRCS	0.613	0.706	0.845	0.721
2	396-412	KSSNILLDEFSTAKVAD	0.582	0.789	0.887	0.753
3	455-471	SFGVVLAELLTGLKPVS	0.754	0.855	0.922	0.844
SlGC34						
1	658-672	KNSLFQILDRRVVRE	0.525	0.801	0.784	0.704
2	277-292	SHCVDSDTGLGGYRCH	0.61	0.701	0.846	0.719
3	97-113	SDSEIRVSNMLYQRCYS	0.593	0.768	0.805	0.722
4	562-578	KSSNILLDNGYTAKVAD	0.569	0.794	0.914	0.759
5	621-637	SFGVVVAELLTGMKPIS	0.782	0.832	0.918	0.844
6	658-674	KNSLFQILDRRVVREGS	0.525	0.801	0.784	0.704
7	277-293	SHCVDSDTGLGGYRCHC	0.61	0.701	0.846	0.719
SlGC35						
1	514-530	KNGAPWLSLENRLRIAS	0.538	0.734	0.839	0.704
SlGC36						
1	634-650	SFGVVLAELLTGIKPIS	0.753	0.855	0.922	0.843
SlGC37						
1	289-304	SQCVDSDTSLGGYRCS	0.598	0.723	0.876	0.732
2	635-651	SFGVVLAELLTGMKPIS	0.768	0.839	0.918	0.842
SlGC38						
1	296-312	KRSGRVSARARLAKARE	0.6	0.71	0.68	0.663
2	524-540	SFGVVLLELLTSQKAVD	0.784	0.873	0.912	0.856
3	296-312	KRSGRVSARARLAKARE	0.6	0.71	0.68	0.663
SlGC39						
1	474-489	KSSNILLDDKLDAKVS	0.52	0.844	0.777	0.713
2	252-268	KSGHYWDAQGYCQKCRH	0.654	0.624	0.878	0.719
3	474-490	KSSNILLDDKLDAKVSD	0.52	0.844	0.777	0.713
4	536-552	SFGVVLLELLTSKKAID	0.786	0.873	0.868	0.842
SlGC40						
1	317-331	SSGGVKSAKLFTGKE	0.672	0.744	0.85	0.755
2	36-51	STGPDCGDQSYKIRCS	0.538	0.8	0.828	0.722
3	464-480	KSSNILLDDKLNAKVAD	0.534	0.844	0.82	0.733
4	523-539	SFGVVLLELLTSQKAID	0.784	0.873	0.912	0.856
5	167-183	STSYMIRVRESGCRAYR	0.661	0.678	0.761	0.7
SlGC42						
1	148-164	SFGVVLVELLSSKRAVD	0.787	0.869	0.869	0.842
SlGC43						
1	631-645	SCSSPRSMSETSFRS	0.565	0.744	0.828	0.712
2	461-477	KSSNILLDYNFNSKVAD	0.622	0.815	0.89	0.776
3	520-536	SFGVVLVEIITAMKVVD	0.72	0.867	0.918	0.835
4	317-333	SNSVHVFQYKEIERATN	0.616	0.764	0.805	0.728
5	637-653	SMSETSFRSTTTKKGVG	0.667	0.777	0.804	0.749
6	649-665	KKGVGSRRLIVPQKIAN	0.686	0.751	0.765	0.734
SlGC44						
1	234-250	SGCRKDETTCNPSKYLS	0.6	0.778	0.765	0.714
2	448-464	KSSNILLDYNYKSKVAD	0.649	0.785	0.855	0.763
3	507-523	SFGVVLAEIITGLKAVD	0.752	0.855	0.921	0.842
SlGC45						

1	616-630	SFGPVYYGRLRDGKE	0.749	0.692	0.764	0.735
2	733-748	KTSNILLDINMRAKVS	0.593	0.837	0.837	0.756
3	130-146	SESSRIYVNEMIIRAPS	0.489	0.806	0.818	0.704
4	733-749	KTSNILLDINMRAKVSD	0.593	0.837	0.837	0.756
5	792-808	SFGVVLLELISGRKPFS	0.768	0.807	0.857	0.811
S1GC46						
1	220-235	STGVMSCIFSIDLKKD	0.66	0.819	0.898	0.792
2	220-236	STGVMSCIFSIDLKKDS	0.66	0.819	0.898	0.792
3	508-524	SFGVVVLEIMCGRKVLD	0.815	0.8	0.848	0.821
S1GC47						
1	266-282	SFGVVLLELLTGRKPVD	0.783	0.822	0.856	0.82
S1GC48						
1	270-286	SFGVVLLELLTGRKPVD	0.783	0.822	0.856	0.82
S1GC49						
1	26-41	KAGNPQSYGAGSERGE	0.658	0.661	0.888	0.735
2	271-287	SFGVVLLELLTGRKPVD	0.783	0.822	0.856	0.82
S1GC50						
1	265-281	SFGVVLLELLTGRKPVD	0.783	0.822	0.856	0.82
S1GC51						
1	168-183	KRGSLDCECVYPLKID	0.654	0.875	0.846	0.792
2	519-535	KASNILLENNFHAKVAD	0.569	0.801	0.898	0.756
S1GC52						
1	294-309	KASNVLLEEDFTPKVS	0.58	0.832	0.849	0.754
2	294-310	KASNVLLEEDFTPKVSD	0.58	0.832	0.849	0.754
3	353-369	SYGVVLLELLSGRKPVD	0.769	0.807	0.857	0.811
S1GC53						
1	764-779	KSSNILLEHDFTPKVS	0.569	0.836	0.847	0.751
2	609-625	SFSSSFAAYTGSARTFS	0.751	0.694	0.945	0.797
3	764-780	KSSNILLEHDFTPKVSD	0.569	0.836	0.847	0.751
4	824-840	SYGVVLLELLTGKKPVD	0.783	0.846	0.872	0.834
S1GC54						
1	460-475	KTSGDAGSMILASKPS	0.65	0.732	0.9	0.761
2	635-650	KSSNILLEHDFTPKVS	0.569	0.836	0.847	0.751
3	460-476	KTSGDAGSMILASKPSS	0.65	0.732	0.9	0.761
4	480-496	SFSSSILAYTGTAKIFS	0.704	0.743	0.942	0.796
5	635-651	KSSNILLEHDFTPKVSD	0.569	0.836	0.847	0.751
6	695-711	SYGVVLLELLSGRKPVD	0.769	0.807	0.857	0.811
S1GC55						
1	594-608	SSSGDSSELDHHQRS	0.566	0.778	0.8	0.715
2	451-467	SFGIVLLELITGKRPID	0.785	0.85	0.872	0.836
S1GC56						
1	522-536	SFGVVLLELITGRKS	0.782	0.822	0.855	0.82
2	676-691	KPGCGDQLHVLLSKID	0.558	0.81	0.879	0.749
3	123-139	SHGNSSPPQSPEPKKGS	0.524	0.838	0.851	0.738
4	522-538	SFGVVLLELITGRKSVD	0.782	0.822	0.855	0.82
5	676-692	KPGCGDQLHVLLSKIDH	0.558	0.81	0.879	0.749
S1GC57						
1	581-595	SFGVVLLELITGRKS	0.782	0.822	0.855	0.82
2	522-538	KSSNILLDNNFDARVAD	0.532	0.846	0.854	0.744
3	581-597	SFGVVLLELITGRKSVD	0.782	0.822	0.855	0.82
S1GC58						

1	541-556	KSSNILLDINFEARVS	0.57	0.834	0.854	0.753
2	541-557	KSSNILLDINFEARVSD	0.57	0.834	0.854	0.753
3	600-616	SFGVVLLELITGRKPVD	0.782	0.822	0.855	0.82
SlGC59						
1	113-128	KPGSGIVIAVKKLKPE	0.616	0.812	0.828	0.752
2	220-235	KASNILLDAEFNSKLS	0.642	0.803	0.848	0.764
3	220-236	KASNILLDAEFNSKLSD	0.642	0.803	0.848	0.764
4	280-296	SFGVVLLELLSGRRAVD	0.769	0.807	0.857	0.811
SlGC60						
1	113-128	KPGSGMVIAVKKLKPE	0.633	0.795	0.832	0.753
2	220-235	KASNILLDGEFNAKLS	0.587	0.775	0.854	0.739
3	220-236	KASNILLDGEFNAKLSD	0.587	0.775	0.854	0.739
4	280-296	SFGVVLLELLSGRRALD	0.769	0.807	0.857	0.811
SlGC61						
1	580-596	SFGVVLVELVTGRKAVD	0.811	0.832	0.856	0.833
SlGC62						
1	120-134	SGSPHGAVAAEAKKS	0.669	0.686	0.828	0.728
2	14-29	SSCDVAEKVMVAVKAS	0.629	0.762	0.822	0.738
3	584-600	SFGVVLVELVTGRKAVD	0.811	0.832	0.856	0.833
4	14-30	SSCDVAEKVMVAVKASN	0.629	0.762	0.822	0.738
SlGC63						
1	267-281	SFGVVLLELLTGRRS	0.783	0.822	0.856	0.82
2	21-36	SESPKDKSPSQNARMD	0.487	0.828	0.77	0.695
3	207-222	KTSNILLDLEYNTKLS	0.626	0.854	0.856	0.779
4	21-37	SESPKDKSPSQNARMDH	0.487	0.828	0.77	0.695
5	207-223	KTSNILLDLEYNTKLSD	0.626	0.854	0.856	0.779
6	267-283	SFGVVLLELLTGRRSLD	0.783	0.822	0.856	0.82
SlGC64						
1	223-238	KTSNILLDSDFTAKLS	0.533	0.84	0.861	0.745
2	223-239	KTSNILLDSDFTAKLSD	0.533	0.84	0.861	0.745
3	283-299	SFGVVLLELLTGRRAMD	0.783	0.822	0.856	0.82
SlGC65						
1	271-285	SFGVVLLELLTGKRS	0.783	0.846	0.872	0.834
2	211-226	KASNILIDSDYTAKLS	0.576	0.801	0.861	0.746
3	211-227	KASNILIDSDYTAKLSD	0.576	0.801	0.861	0.746
4	271-287	SFGVVLLELLTGKRSLD	0.783	0.846	0.872	0.834
SlGC66						
1	217-232	KASNILLDSDYRAKLS	0.592	0.762	0.8	0.718
2	217-233	KASNILLDSDYRAKLSD	0.592	0.762	0.8	0.718
3	277-293	SFGVVLLELITGRRAMD	0.782	0.822	0.855	0.82
SlGC67						
1	270-284	SFGVVLLELLTGRRS	0.783	0.822	0.856	0.82
2	210-225	KASNILLDSDYTAKLS	0.577	0.801	0.862	0.747
3	210-226	KASNILLDSDYTAKLSD	0.577	0.801	0.862	0.747
4	270-286	SFGVVLLELLTGRRSVD	0.783	0.822	0.856	0.82
SlGC68						
1	263-277	SFGVVLLELLTGRRS	0.783	0.822	0.856	0.82
2	19-34	KTSKLVSKQSTFHRIS	0.61	0.778	0.82	0.736
3	203-218	KASNILLDSDYNAKLS	0.6	0.799	0.854	0.751
4	203-219	KASNILLDSDYNAKLSD	0.6	0.799	0.854	0.751
5	263-279	SFGVVLLELLTGRRSVD	0.783	0.822	0.856	0.82

6	19-35	KTSKLVSKQSTFHRISA	0.61	0.778	0.82	0.736
S1GC69						
1	444-458	KKSPVIIEGHNRTRS	0.606	0.792	0.767	0.722
2	230-245	KGSNILLGEGYHPKLS	0.633	0.712	0.911	0.752
3	230-246	KGSNILLGEGYHPKLSD	0.633	0.712	0.911	0.752
4	290-306	SFGVVLLEIITGRKAID	0.781	0.822	0.855	0.819
5	444-460	KKSPVIIEGHNRTRSID	0.606	0.792	0.767	0.722
S1GC70						
1	230-245	KCSNILLGEGFHPKLS	0.629	0.763	0.907	0.766
2	230-246	KCSNILLGEGFHPKLSD	0.629	0.763	0.907	0.766
3	290-306	SFGVVLLEIITGRRAID	0.781	0.822	0.855	0.819
S1GC71						
1	212-227	KSSNILLKENFFPKLS	0.517	0.813	0.858	0.73
2	212-228	KSSNILLKENFFPKLSD	0.517	0.813	0.858	0.73
3	272-288	SFGVVFLELITGRKAID	0.78	0.79	0.862	0.811
S1GC72						
1	409-423	SPSIQRNSPDSRKRD	0.573	0.729	0.72	0.674
2	214-229	KSSNILLDEGYHPKLS	0.589	0.78	0.885	0.751
3	409-424	SPSIQRNSPDSRKRDS	0.573	0.729	0.72	0.674
4	446-461	KWGVDESERPDSQRNS	0.569	0.819	0.745	0.711
5	214-230	KSSNILLDEGYHPKLSD	0.589	0.78	0.885	0.751
6	274-290	SFGVVFLELITGRKAID	0.78	0.79	0.862	0.811
S1GC73						
1	54-68	KSGESNNQNNNVARS	0.455	0.821	0.921	0.733
2	208-223	KSSNILLDNDFNPKLS	0.55	0.869	0.861	0.76
3	208-224	KSSNILLDNDFNPKLSD	0.55	0.869	0.861	0.76
4	268-284	SFGVVLLELITGRKAYD	0.782	0.822	0.855	0.82
S1GC74						
1	476-490	KSSSRAIRSTSRRKS	0.655	0.636	0.683	0.658
2	493-507	KRSESIISNDDTEKD	0.515	0.877	0.714	0.702
3	274-290	SFGVVLLEIITGRRAVD	0.781	0.822	0.855	0.819
4	467-483	SESKRKSKIKSSSRAIR	0.569	0.784	0.63	0.661
S1GC75						
1	735-750	KISIWCIQDEPSLRPS	0.616	0.777	0.883	0.759
2	677-693	SFGVVLLELICRRKCVD	0.796	0.823	0.785	0.801
S1GC76						
1	183-197	SLGSLEDHLLDLERD	0.59	0.838	0.788	0.739
2	234-249	KSSNILLDKEHNAKLS	0.578	0.83	0.787	0.732
3	234-250	KSSNILLDKEHNAKLSD	0.578	0.83	0.787	0.732
4	294-310	SFGVVLLELITGRRAVD	0.782	0.822	0.855	0.82
S1GC77						
1	228-243	KSSNILLDKEYNAKLS	0.578	0.803	0.813	0.731
2	228-244	KSSNILLDKEYNAKLSD	0.578	0.803	0.813	0.731
3	288-304	SFGVVLLELITGKRAVD	0.782	0.846	0.872	0.833
S1GC78						
1	107-122	KKSAFGIYDMCLIRYS	0.588	0.732	0.862	0.727
2	221-236	KRGNYHLMPSCVVRYE	0.555	0.82	0.833	0.736
3	468-484	KASNVLLDAEMNPKIAD	0.666	0.829	0.86	0.785
4	107-123	KKSAFGIYDMCLIRYSN	0.588	0.732	0.862	0.727
S1GC79						
1	249-263	SHSPPPGRSAFAGKE	0.534	0.733	0.837	0.701

2	249-264	SHSPPPGRSAFAGKED	0.534	0.733	0.837	0.701
3	459-474	KASNVLLDAEMNPKIS	0.666	0.829	0.86	0.785
4	459-475	KASNVLLDAEMNPKISD	0.666	0.829	0.86	0.785
5	249-265	SHSPPPGRSAFAGKEDK	0.534	0.733	0.837	0.701
SlGC80						
1	219-234	KIGGRIIGPRCNFRYE	0.601	0.686	0.769	0.686
2	474-490	KAGNVLLDAEMNPKIAD	0.666	0.829	0.86	0.785
3	219-235	KIGGRIIGPRCNFRYEI	0.601	0.686	0.769	0.686
4	534-550	SFGVLVLEIISGQRNIC	0.766	0.824	0.917	0.836
SlGC81						
1	221-236	KIGGRIIGVRCNFRYE	0.652	0.688	0.776	0.706
2	469-484	KASNVLLDAEMNPKIS	0.666	0.829	0.86	0.785
3	469-485	KASNVLLDAEMNPKISD	0.666	0.829	0.86	0.785
4	221-237	KIGGRIIGVRCNFRYEI	0.652	0.688	0.776	0.706
SlGC82						
1	485-500	KASNVLLDKDMNAKIS	0.592	0.838	0.803	0.744
2	485-501	KASNVLLDKDMNAKISD	0.592	0.838	0.803	0.744
3	545-561	SFGVLLLEIVSGRKNNS	0.768	0.806	0.857	0.81
SlGC83						
1	568-584	KASNVLLDENMNPKIAD	0.621	0.861	0.877	0.786
2	628-644	SFGVLMLEILSGRRNAS	0.771	0.79	0.86	0.807
SlGC84						
1	435-451	KLGEGGFGPVYKGKFPD	0.68	0.645	0.856	0.727
2	557-573	KASNVLLDENMNPKIAD	0.621	0.861	0.877	0.786
3	617-633	SFGILMLEIVSGRRTTS	0.786	0.793	0.86	0.813
SlGC85						
1	595-610	KASNVLLDDNMNPKIS	0.605	0.874	0.872	0.784
2	595-611	KASNVLLDDNMNPKISD	0.605	0.874	0.872	0.784
3	655-671	SFGVLVLEILSGKRNNS	0.769	0.824	0.873	0.822
SlGC86						
1	171-185	KTGQVWSLVSWISKD	0.622	0.742	0.938	0.767
2	618-633	KASNILLDAEMNPKIS	0.653	0.828	0.859	0.78
3	618-634	KASNILLDAEMNPKISD	0.653	0.828	0.859	0.78
4	678-694	SFGVLLLEIVSGKKNNS	0.767	0.83	0.873	0.824
SlGC87						
1	32-47	SFSAANTLVSKQGKFE	0.703	0.825	0.893	0.807
2	622-637	KASNILLDEDMNPKIS	0.593	0.86	0.838	0.763
3	95-111	SDGNLGIFNALKQRVWD	0.607	0.772	0.86	0.747
4	622-638	KASNILLDEDMNPKISD	0.593	0.86	0.838	0.763
5	682-698	SFGVILLEIISGKKNRI	0.753	0.831	0.872	0.819
SlGC88						
1	43-57	SAGNIFELGFFSPRS	0.646	0.729	0.908	0.761
2	43-58	SAGNIFELGFFSPRSS	0.646	0.729	0.908	0.761
3	534-549	KIGAGGFGPVYKGKLE	0.714	0.63	0.897	0.747
4	679-694	KASNILLDDDMNPKIS	0.577	0.873	0.832	0.761
5	739-754	SFGVLLLEIVSGKRNS	0.767	0.83	0.873	0.824
6	1397-1412	KIGAGGFGPVYKGKLE	0.714	0.63	0.897	0.747
7	1519-1534	KASNVLLDDEMNPKIS	0.603	0.861	0.839	0.768
8	1579-1594	SFGVLLLEIVSGKKNS	0.767	0.83	0.873	0.824
9	534-550	KIGAGGFGPVYKGKLED	0.714	0.63	0.897	0.747
10	679-695	KASNILLDDDMNPKISD	0.577	0.873	0.832	0.761

11	1397-1413	KIGAGGFGPVYKGKLED	0.714	0.63	0.897	0.747
12	1519-1535	KASNVLLDDEMNPKISD	0.603	0.861	0.839	0.768
13	43-59	SAGNIFELGFFSPRSSR	0.646	0.729	0.908	0.761
SlGC89						
1	511-526	KIGAGGFGPVYKGKLE	0.714	0.63	0.897	0.747
2	633-648	KASNILLDGDMNPKIS	0.613	0.828	0.853	0.765
3	511-527	KIGAGGFGPVYKGKLED	0.714	0.63	0.897	0.747
4	633-649	KASNILLDGDMNPKISD	0.613	0.828	0.853	0.765
5	693-709	SFGVLLLEIVSGQKNNS	0.766	0.83	0.918	0.838
SlGC90						
1	319-334	STSTCDCLSGFKPKFE	0.641	0.821	0.871	0.778
2	654-669	KTSNILLDEELNPKIS	0.573	0.863	0.843	0.76
3	654-670	KTSNILLDEELNPKISD	0.573	0.863	0.843	0.76
4	319-335	STSTCDCLSGFKPKFEK	0.641	0.821	0.871	0.778
SlGC91						
1	669-684	KASNILLDKEMIPKIS	0.541	0.849	0.821	0.737
2	402-418	SGCVIWTKELVDMRQYS	0.667	0.739	0.82	0.742
3	669-685	KASNILLDKEMIPKISD	0.541	0.849	0.821	0.737
SlGC92						
1	321-335	SKGCARKTSLNCQKE	0.643	0.804	0.747	0.731
2	790-804	SQSTATEADTSSSKH	0.656	0.788	0.895	0.78
3	627-642	KPSNVLLDTDMNPKIS	0.589	0.888	0.848	0.775
4	722-737	KESRVMEVIDEQLRQS	0.561	0.832	0.741	0.711
5	627-643	KPSNVLLDTDMNPKISD	0.589	0.888	0.848	0.775
6	790-806	SQSTATEADTSSSKHGE	0.656	0.788	0.895	0.78
7	159-175	KTGFRGFLRSWKSRNNP	0.642	0.626	0.758	0.675
SlGC93						
1	264-279	SNGVLQRSTWDENRQE	0.686	0.75	0.798	0.744
2	618-633	KASNILLDADMNPKIS	0.64	0.841	0.852	0.778
3	618-634	KASNILLDADMNPKISD	0.64	0.841	0.852	0.778
4	264-280	SNGVLQRSTWDENRQEW	0.686	0.75	0.798	0.744
SlGC94						
1	234-249	SFGVVINSREIYYKYE	0.615	0.762	0.845	0.741
2	638-653	KASNVLLDIEMNPKIS	0.646	0.862	0.86	0.789
3	394-410	SGCLIWIDELIDIRQLS	0.594	0.776	0.842	0.738
4	638-654	KASNVLLDIEMNPKISD	0.646	0.862	0.86	0.789
SlGC95						
1	232-247	SFGLVMNNKEIYYKYE	0.612	0.794	0.863	0.756
2	638-653	KASNVLLDTDMNPKIS	0.634	0.869	0.855	0.786
3	392-408	SGCLIWIDELIDIRQLS	0.594	0.776	0.842	0.738
4	638-654	KASNVLLDTDMNPKISD	0.634	0.869	0.855	0.786
SlGC96						
1	40-55	SDGTFELGFFSAGKNS	0.624	0.661	0.892	0.726
2	55-70	SSSRNRYIGIWYKKIS	0.588	0.608	0.756	0.651
3	232-247	SFGLVMNDQEIYYKYE	0.6	0.793	0.88	0.758
4	636-651	KASNVLLDFEMNPKIS	0.645	0.83	0.867	0.781
5	40-56	SDGTFELGFFSAGKNSS	0.624	0.661	0.892	0.726
6	392-408	SGCLLWIGELVDLRQLS	0.656	0.716	0.874	0.749
7	636-652	KASNVLLDFEMNPKISD	0.645	0.83	0.867	0.781
SlGC97						
1	332-346	SNGCVRRKPFDCNKE	0.586	0.781	0.681	0.682

2	332-347	SNGCVRRKPFDCNKEH	0.586	0.781	0.681	0.682
3	618-633	KASNILLDMEMNPKIS	0.649	0.845	0.862	0.786
4	618-634	KASNILLDMEMNPKISD	0.649	0.845	0.862	0.786
5	678-694	SFGVLVLETVSCKKNRS	0.756	0.858	0.86	0.825
SlGC98						
1	621-636	KASNVLLDTDLNPKIS	0.616	0.864	0.852	0.777
2	398-414	KGCLLWFGELIDIRKLS	0.636	0.704	0.881	0.74
3	621-637	KASNVLLDTDLNPKISD	0.616	0.864	0.852	0.777
SlGC99						
1	334-348	SQGCVRKTSIDCNKE	0.643	0.842	0.762	0.749
2	399-413	KGCLFWSGELIDIRE	0.641	0.709	0.874	0.741
3	334-349	SQGCVRKTSIDCNKEH	0.643	0.842	0.762	0.749
4	340-355	KTSIDCNKEHGFVKYS	0.539	0.752	0.815	0.702
5	621-636	KASNVLLDTEMNPKIS	0.648	0.857	0.862	0.789
6	399-415	KGCLFWSGELIDIRELS	0.641	0.709	0.874	0.741
7	621-637	KASNVLLDTEMNPKISD	0.648	0.857	0.862	0.789

Table S3 List of 99 GC-kinases identified in tomato genome

SlGCs	Protein ID	Protein Domain	Position of GC CC
SlGC1	Solyc02g084370.1.1	LRR-NT, LRR, Pkinase	850-865
SlGC2	Solyc03g033610.1.1	LRR-NT, LRR, Pkinase	1032-1046
SlGC3	Solyc04g081080.1.1	LRR-NT, LRR, Pkinase	1091-1106
SlGC4	Solyc02g067560.1.1	LRR, Pkinase	488-504
SlGC5	Solyc02g087460.1.1	LRR-NT, LRR, Pkinase_Tyr	485-501
SlGC6	Solyc04g008430.1.1	LRR-NT, LRR, Pkinase	1021-1037
SlGC7	Solyc01g103530.2.1	LRR-NT, LRR, Pkinase	870-886
SlGC8	Solyc01g080770.2.1	LRR-NT, LRR, Pkinase	842-858
SlGC9	Solyc02g091840.2.1	LRR-NT, LRR, Pkinase_Tyr	878-894
SlGC10	Solyc04g081590.2.1	LRR-NT, LRR, Pkinase_Tyr	881-897
SlGC11	Solyc09g064520.2.1	LRR-NT, LRR, Pkinase	898-914
SlGC12	Solyc03g093330.2.1	LRR-NT, LRR, Pkinase	863-879
SlGC13	Solyc05g051640.2.1	LRR-NT, LRR, Pkinase	901-917
SlGC14	Solyc02g091860.2.1	LRR-NT, LRR, Pkinase	876-892
SlGC15	Solyc07g053600.2.1	LRR-NT, LRR, Pkinase	879-895
SlGC16	Solyc02g077630.2.1	LRR-NT, LRR, Pkinase	886-902
SlGC17	Solyc03g112580.2.1	LRR-NT, LRR, Pkinase	954-970
SlGC18	Solyc03g123860.2.1	LRR-NT, LRR, Pkinase	1005-1021
SlGC19	Solyc04g076990.2.1	Pkinase	411-427
SlGC20	Solyc11g020280.1.1	LRR-NT, LRR, Pkinase	872-888
SlGC21	Solyc06g065260.2.1	LRR-NT, LRR, Pkinase	869-885
SlGC22	Solyc08g061560.2.1	LRR-NT, LRR, Pkinase	843-859
SlGC23	Solyc03g007050.2.1	LRR-NT, LRR, Pkinase	837-853
SlGC24	Solyc07g063000.2.1	LRR-NT, LRR, Pkinase	924-940
SlGC25	Solyc01g008140.2.1	LRR, Pkinase	871-887
SlGC26	Solyc07g062040.2.1	Pkinase	222-238
SlGC27	Solyc03g059490.1.1	LRR-NT, LRR, Pkinase	1031-1047
SlGC28	Solyc04g005390.1.1	LRR-NT, LRR, Pkinase	638-654
SlGC29	Solyc01g111950.2.1	Pkinase	435-451
SlGC30	Solyc01g108000.2.1	Pkinase	514-530
SlGC31	Solyc02g068660.1.1	Pkinase	565-581
SlGC32	Solyc09g015240.1.1	WAK, Ca^{2+} _binding EGF, Pkinase	300-315
SlGC33	Solyc10g076530.1.1	Ca^{2+} _binding EGF, Pkinase	455-471
SlGC34	Solyc09g014740.2.1	Ca^{2+} _binding EGF, Pkinase	621-637
SlGC35	Solyc09g014730.2.1	Ca^{2+} _binding EGF, Pkinase	514-530
SlGC36	Solyc09g014710.2.1	Ca^{2+} _binding EGF, Pkinase	634-650
SlGC37	Solyc09g014720.1.1	Ca^{2+} _binding EGF, Pkinase	635-651
SlGC38	Solyc09g008640.1.1	Pkinase	524-540
SlGC39	Solyc09g011200.1.1	Pkinase	536-552
SlGC40	Solyc12g088040.1.1	Pkinase	523-539
SlGC41	Solyc10g076560.1.1	Pkinase	119-135
SlGC42	Solyc05g009010.1.1	Pkinase	148-164
SlGC43	Solyc02g090110.2.1	Pkinase	520-536
SlGC44	Solyc04g079710.2.1	Pkinase	507-523
SlGC45	Solyc05g014240.2.1	LRR, Pkinase	792-808
SlGC46	Solyc01g104050.2.1	Pkinase	508-524

SlGC47	Solyc10g080580.1.1	Pkinase_Tyr	266-282
SlGC48	Solyc10g078940.1.1	Pkinase_Tyr	270-286
SlGC49	Solyc03g059080.1.1	Pkinase_Tyr	271-287
SlGC50	Solyc12g098820.1.1	Pkinase_Tyr	265-281
SlGC51	Solyc01g079340.2.1	Pkinase	519-535
SlGC52	Solyc09g064270.2.1	Pkinase_Tyr	353-369
SlGC53	Solyc07g039340.2.1	Pkinase_Tyr	824-840
SlGC54	Solyc03g121610.2.1	Pkinase_Tyr	695-711
SlGC55	Solyc02g085430.2.1	Pkinase_Tyr	451-467
SlGC56	Solyc04g006930.2.1	Pkinase	522-538
SlGC57	Solyc01g010030.2.1	Pkinase	581-597
SlGC58	Solyc05g010140.2.1	Pkinase	600-616
SlGC59	Solyc05g007140.2.1	Pkinase_Tyr	280-296
SlGC60	Solyc01g010660.2.1	Pkinase_Tyr	280-296
SlGC61	Solyc03g121440.2.1	Pkinase_Tyr	580-596
SlGC62	Solyc12g044840.1.1	Pkinase	584-600
SlGC63	Solyc01g028830.2.1	Pkinase_Tyr	267-283
SlGC64	Solyc08g074980.2.1	Pkinase_Tyr	283-299
SlGC65	Solyc08g061250.2.1	Pkinase_Tyr	271-287
SlGC66	Solyc06g062920.2.1	Pkinase_Tyr	277-293
SlGC67	Solyc07g041940.2.1	Pkinase_Tyr	270-286
SlGC68	Solyc05g025820.2.1	Pkinase_Tyr	263-279
SlGC69	Solyc11g072660.1.1	Pkinase	290-306
SlGC70	Solyc06g075550.2.1	Pkinase	290-306
SlGC71	Solyc05g024290.2.1	Pkinase	272-288
SlGC72	Solyc01g067400.2.1	Pkinase	274-290
SlGC73	Solyc12g094680.1.1	Pkinase	268-284
SlGC74	Solyc04g050970.2.1	Pkinase	274-290
SlGC75	Solyc11g005630.1.1	D-mannose binding lectin, S-locus glycoprotein, Pkinase	677-693
SlGC76	Solyc09g061330.1.1	Pkinase	294-310
SlGC77	Solyc06g083500.2.1	Pkinase	288-304
SlGC78	Solyc02g080010.2.1	Salt stress response/antifungal, Pkinase	468-484
SlGC79	Solyc02g079990.2.1	Salt stress response/antifungal, Pkinase_tyr	459-475
SlGC80	Solyc02g080080.2.1	Salt stress response/antifungal, Pkinase	534-550
SlGC81	Solyc02g080070.2.1	Salt stress response/antifungal, Pkinase_tyr	469-485
SlGC82	Solyc03g111540.1.1	Salt stress response/antifungal, Pkinase_tyr	545-561
SlGC83	Solyc08g076060.2.1	D-mannose binding lectin, PAN-like, Pkinase_Tyr	628-644
SlGC84	Solyc08g076050.2.1	D-mannose binding lectin, PAN-like, Pkinase_Tyr	617-633
SlGC85	Solyc03g120110.2.1	D-mannose binding lectin, PAN-like, Pkinase_Tyr	655-671
SlGC86	Solyc12g006840.1.1	D-mannose binding lectin, PAN-like, Pkinase_Tyr	678-694
SlGC87	Solyc02g079710.2.1	D-mannose binding lectin, S-locus	682-698

		glycoprotein, PAN-like, Pkinase_Tyr	
SlGC88	Solyc03g006730.1.1	D-mannose binding lectin, S-locus glycoprotein, PAN-like, Pkinase_Tyr	739-754
SlGC89	Solyc03g006720.1.1	D-mannose binding lectin, S-locus glycoprotein, PAN-like, Pkinase_Tyr	693-709
SlGC90	Solyc05g008310.2.1	D-mannose binding lectin, S-locus glycoprotein, PAN-like, Pkinase_Tyr	654-670
SlGC91	Solyc03g006770.2.1	D-mannose binding lectin, S-locus glycoprotein, PAN-like, Pkinase_Tyr	669-685
SlGC92	Solyc02g030300.2.1	D-mannose binding lectin, S-locus glycoprotein, PAN-like, Pkinase_Tyr	627-643
SlGC93	Solyc04g077390.2.1	D-mannose binding lectin, S-locus glycoprotein, PAN-like, Pkinase_Tyr	618-634
SlGC94	Solyc07g063770.2.1	D-mannose binding lectin, S-locus glycoprotein, PAN-like, Pkinase_Tyr	638-654
SlGC95	Solyc07g063820.2.1	D-mannose binding lectin, S-locus glycoprotein, PAN-like, Pkinase_Tyr	638-654
SlGC96	Solyc10g006710.2.1	D-mannose binding lectin, S-locus glycoprotein, PAN-like, Pkinase_Tyr	636-652
SlGC97	Solyc04g077280.2.1	D-mannose binding lectin, S-locus glycoprotein, PAN-like, Pkinase_Tyr	678-694
SlGC98	Solyc04g077360.2.1	D-mannose binding lectin, S-locus glycoprotein, PAN-like, Pkinase_Tyr	621-637
SlGC99	Solyc04g077270.2.1	D-mannose binding lectin, S-locus glycoprotein, Pkinase_Tyr	621-637

A

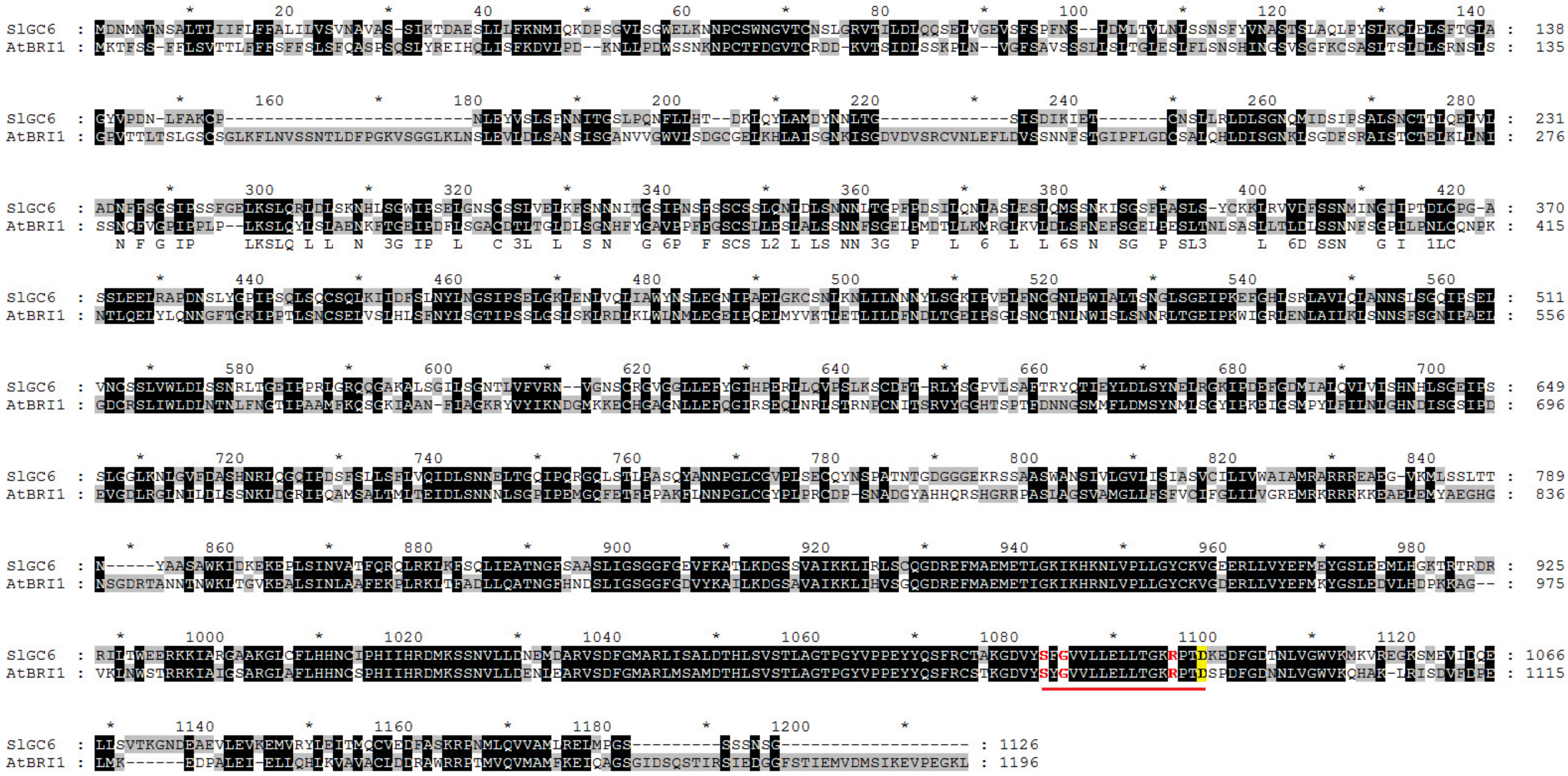

B

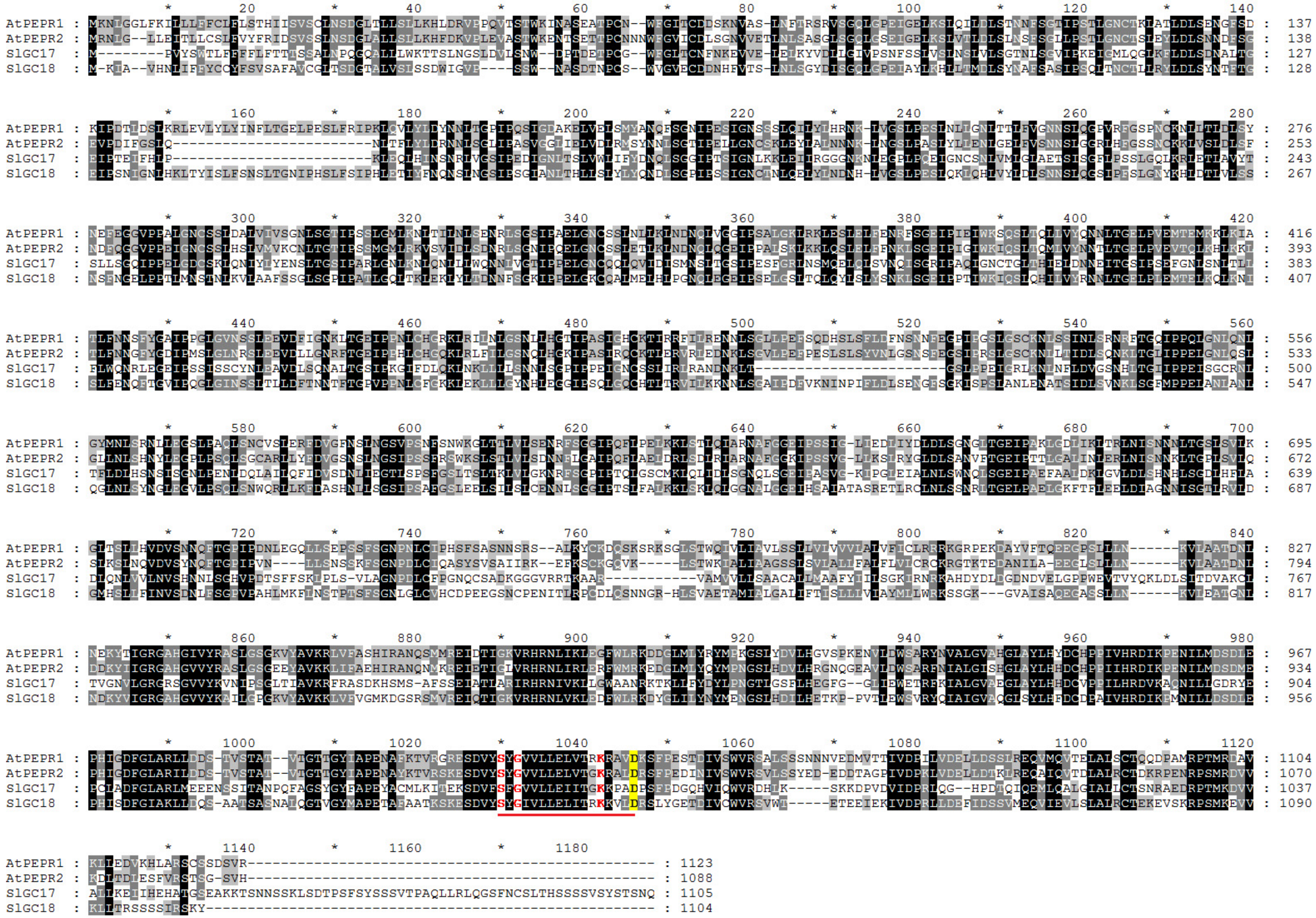

C

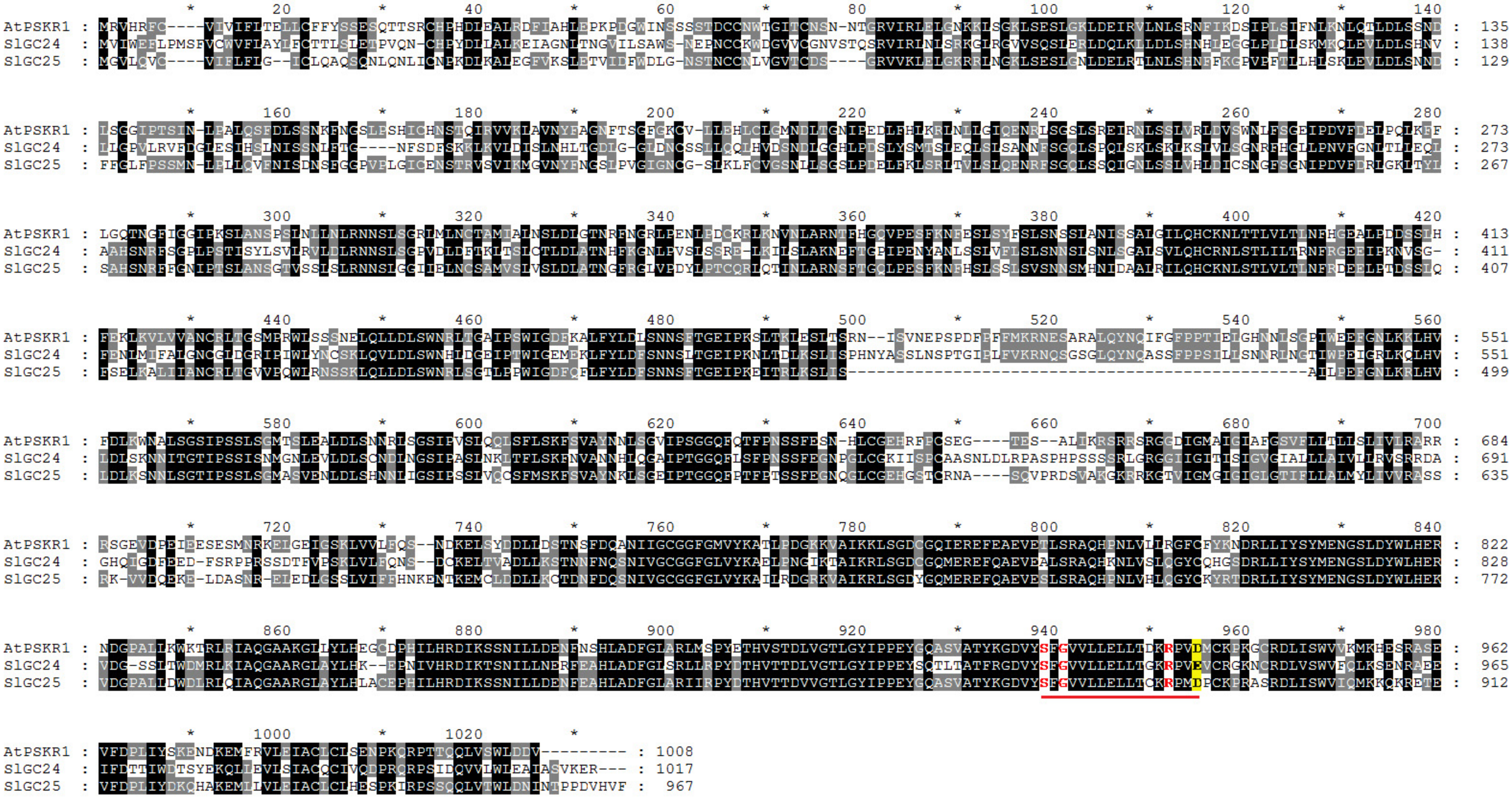

Figure S1. Alignment of amino acid sequences of Arabidopsis GCs and their tomato orthologs. (A) Alignment of AtBRI1 with SlGC6. (B) Alignment of AtPEPRs with SlGC17 and SlGC18. (C) Alignment of AtPSKR1 with SlGC24 and SlGC25. The 14 amino acid residues of GC catalytic center motif are underlined and the amino acids required for GC catalysis function are highlighted in colors.
